# Structural Mechanism of Trimeric HIV-1 Envelope Glycoprotein Activation

**DOI:** 10.1371/journal.ppat.1002797

**Published:** 2012-07-12

**Authors:** Erin E. H. Tran, Mario J. Borgnia, Oleg Kuybeda, David M. Schauder, Alberto Bartesaghi, Gabriel A. Frank, Guillermo Sapiro, Jacqueline L. S. Milne, Sriram Subramaniam

**Affiliations:** 1 Laboratory of Cell Biology, Center for Cancer Research, National Cancer Institute, NIH, Bethesda, Maryland, United States of America; 2 Department of Electrical and Computer Engineering, University of Minnesota, Minneapolis, Minnesota, United States of America; Institut Pasteur, France

## Abstract

HIV-1 infection begins with the binding of trimeric viral envelope glycoproteins (Env) to CD4 and a co-receptor on target T-cells. Understanding how these ligands influence the structure of Env is of fundamental interest for HIV vaccine development. Using cryo-electron microscopy, we describe the contrasting structural outcomes of trimeric Env binding to soluble CD4, to the broadly neutralizing, CD4-binding site antibodies VRC01, VRC03 and b12, or to the monoclonal antibody 17b, a co-receptor mimic. Binding of trimeric HIV-1 BaL Env to either soluble CD4 or 17b alone, is sufficient to trigger formation of the open quaternary conformation of Env. In contrast, VRC01 locks Env in the closed state, while b12 binding requires a partial opening in the quaternary structure of trimeric Env. Our results show that, despite general similarities in regions of the HIV-1 gp120 polypeptide that contact CD4, VRC01, VRC03 and b12, there are important differences in quaternary structures of the complexes these ligands form on native trimeric Env, and potentially explain differences in the neutralizing breadth and potency of antibodies with similar specificities. From cryo-electron microscopic analysis at ∼9 Å resolution of a cleaved, soluble version of trimeric Env, we show that a structural signature of the open Env conformation is a three-helix motif composed of α-helical segments derived from highly conserved, non-glycosylated N-terminal regions of the gp41 trimer. The three N-terminal gp41 helices in this novel, activated Env conformation are held apart by their interactions with the rest of Env, and are less compactly packed than in the post-fusion, six-helix bundle state. These findings suggest a new structural template for designing immunogens that can elicit antibodies targeting HIV at a vulnerable, pre-entry stage.

## Author Summary

HIV infection occurs following the binding of viral envelope glycoproteins (Env) to receptors on target cell surfaces. Binding to these molecules induces conformational changes in Env, ultimately leading to the exposure of a viral fusion peptide and fusion of viral and cellular membranes. Understanding the structure of Env at each step during HIV entry is of fundamental importance in the design of compounds that can combat infection. Here, we use cryo-electron tomography to characterize the conformational changes that occur in Env at individual steps in the entry process, revealing the unexpected finding that binding to a co-receptor mimic alone induces the same conformational changes as binding to CD4. Furthermore, using single particle cryo-electron microscopy, we show structural evidence, at sub-nanometer resolution, of a novel, activated intermediate state of HIV where highly conserved, interior components of the viral spike are exposed. We show that transition to this state can be blocked by addition of a highly neutralizing antibody, VRC01, revealing a possible mechanism for its potent neutralizing ability. Discovery of the structure of this new Env intermediate provides a template for the design of immunogens aimed at eliciting antibodies that could block HIV entry.

## Introduction

HIV/AIDS is a global health epidemic. Over 2 million people die annually from the disease, which is caused by human immunodeficiency virus (HIV) infection. HIV-1 entry into target cells is initiated by the interaction of the surface envelope glycoproteins (Env) with CD4 and a co-receptor (typically CCR5 or CXCR4) on target cells [1]. Env is a heterodimer of a transmembrane glycoprotein (gp41) and a surface glycoprotein (gp120); these dimers are organized as trimers on the surface of the viral membrane. The gp120 portion of Env is recognized by target cell receptors, including CD4 and a co-receptor molecule, while gp41 promotes fusion of viral and cellular membranes, resulting in viral infection of the cell [2]. Upon binding to the CD4 receptor, gp120 undergoes a conformational change [3], [4], resulting in exposure of epitopes that can be bound by co-receptor molecules [5] and the eventual formation of the transient “pre-hairpin intermediate” conformation. In the “pre-hairpin intermediate”, the gp41 molecules rearrange so that the N-terminal peptides form a trimer of helices that present the fusion peptide to the target cell, while the C-terminal helices remain attached to the viral membrane [2]. This stage is vulnerable to a number of neutralizing antibodies [6] and peptides [7], [8], [9] capable of binding either the N- or C-terminal peptides. Upon fusion with the target cell membrane, further gp41 rearrangement results in the pairing of N- and C-terminal peptides to create a six-helix post-fusion bundle [10]. However, a detailed structural understanding of these molecular rearrangements in the context of native trimeric Env remains elusive.

A number of X-ray crystallographic structures have been reported for selected monomeric gp120 constructs [11], [12], [13], [14], [15], [16], [17], while structural information on gp41 has come from crystallographic and NMR spectroscopic structures of the post-fusion six-helix bundle conformation [18], [19], [20]. Cryo-electron tomographic studies at ∼20 Å resolution have begun to bridge the gap between structures of monomeric gp120 and the structure of native trimeric Env, as displayed on the surface of intact HIV-1 and SIV, and in soluble versions of trimeric Env [21], [22], [23], [24]. These analyses have provided insights into the structures of trimeric Env complexed with neutralizing antibodies and have suggested a working model for structural changes in trimeric Env that occur upon engagement of the CD4 receptor on a target cell [21], [23].

Binding of Env to the target cell CD4 receptor and co-receptor is prevented by antibodies that target the viral CD4 and co-receptor binding sites. Some of the most potent and broadly neutralizing antibodies isolated to date target the CD4 binding site, underscoring the importance of this step in the HIV entry process. The recently described CD4 binding site antibodies VRC01, VRC02, and VRC03 [25] represent a growing family of antibodies, including b12, PG9, PG16, HJ16 and the PGT antibodies, that have the ability to block infection *in vitro* by a broad spectrum of HIV-1 strains [26], [27], [28], [29]. Crystallographic studies of the complexes formed between monomeric HIV-1 gp120 and Fab fragments from antibodies b12 [17], VRC01 [16] or VRC03 (PDB ID:3SE8) have provided important insights into key interactions that underlie antibody recognition of the CD4-binding region of HIV-1 gp120. These antibodies vary in effectiveness: some, such as VRC01, can efficiently neutralize a large proportion (∼91%) of HIV-1 isolates tested, while others, such as b12, neutralize a smaller set (∼41%) of viruses in the same study [25].

A structural understanding of changes in the conformation of trimeric Env at different stages of viral entry and the mechanisms by which antibodies and other reagents block viral entry is critical for the development of effective vaccines and therapeutic agents against HIV/AIDS. Here, we extend our previous cryo-electron tomographic studies [21], [22], [23], [24] and present a more complete picture of the HIV entry process by showing that HIV-1 Env binding to either soluble CD4 (sCD4) or the co-receptor mimic 17b (each representing a distinct step in the HIV entry process), leads to the same structural opening, or activation, of the Env spike. We also demonstrate structurally that the broadly neutralizing antibodies VRC01, VRC02, VRC03 are able to block this activation, locking Env in a state that resembles closed, native Env. Lastly, we present, at ∼9 Å resolution, the cryo-electron microscopic structure of soluble trimeric Env in the 17b-bound state, revealing it as a novel, activated intermediate conformation of trimeric Env that could serve as a new template for immunogen design.

## Materials and Methods

### Virus, antibodies and soluble Env trimers

HIV-1 BaL virus treated with 2,2′-dithiodipyridine (AT-2), a gift from Julian Bess and Dr. Jeffrey Lifson, was prepared as described previously [30]. Antibodies b12 and 17b were provided by the NIH AIDS Research and Reference Reagent Program (Germantown, MD). Antibodies VRC01, VRC02 and VRC03 were provided by Dr. John Mascola (Vaccine Research Center, NIH, Bethesda, MD). VRC02, 17b and b12 Fab fragments were produced by papain digestion (Southern Biotech, Birmingham, AL). Soluble KNH1144 Env trimers were provided by Drs. Kenneth Kang and William Olson (Progenics Inc.) and were from the same lot used in previously reported cryo-electron tomographic studies [23].

### Preparation of grids

Purified AT-2-treated HIV-1 BaL viruses, with either antibodies, Fab fragments or 2-domain (1–183) soluble CD4 (sCD4), were deposited on Quantifoil MultiA carbon grids and plunge-frozen in liquid ethane maintained at about −180°C to prepare vitrified specimens for cryo-electron tomography. For each experiment, equal volumes of antibody in 1× PBS (∼1 mg/ml) or sCD4 (∼8 mg/ml) and virus in 1× TNE (∼500–1000 mg/ml p24^CA^) were incubated together for 1 hr at 4°C (approximate ratio of 3.3–6.6 mg p24^CA^ to 6 mg of antibody). Immediately before grid preparation, 1.5–2 ml of 10 nm protein A gold was added to the mixture. For each grid, 2 µl was placed on a plasma-cleaned 200-mesh Quantifoil Multi-A grid (Quantifoil, Jena, Germany). Excess buffer was blotted onto filter paper using a Vitrobot Mark III (FEI) for six seconds at an offset of −2 mm. Blotting was done at room temperature in 100% humidity. Grids were immediately plunged into liquid ethane and stored in liquid N2 before imaging.

### Cryo electron microscopy

Specimens for tomography were imaged in a Polara Tecnai G2 transmission electron microscope (FEI) operated at 200 kV and equipped with an energy filter (Gatan), with the specimen maintained at liquid nitrogen temperatures. Typically, ∼61 images were taken for each tilt series in 2° increments between ±60° at an average dose of ∼2.5 electrons/Å^2^ for each image. Tilt series were imaged at 34,000× with a defocus value of ∼2.5 µm using a camera with a pixel size of 4.1 Å. For single particle analysis, data were collected in an FEI Titan Krios microscope (FEI Company, Hillsboro, OR) operated at 80 kV. Images were recorded using a Gatan US4000 4K×4K CCD camera (Gatan Inc. Pleasanton, CA), at a nominal magnification of 75,000×, corresponding to a pixel size of 1.08 A. A total of 3,299 micrographs (4096×4096 pixel) were collected at underfocus values spanning the range from 1.5 to 5 µm.

### Tomographic image analysis

Fiducial-based reconstruction of tomograms was carried out using weighted back-projection techniques [31]. The 3D reconstructions presented were derived typically from about 60 tilt series (range 34–95) with spikes from about 300 individual virions (range 177–408) contributing to the final map. Automatic fiducial-based reconstruction of tomograms was performed using IMOD software. Individual virions were segmented and subjected to automated spike-picking procedures. Alignment, classification and 3D averaging of the extracted spike volumes was done as previously described [32] for experiments with bound VRC01, VRC02 or VRC03 antibodies, and final density maps were obtained after five to seven 3D classification and alignment iterations beginning with the raw spike images. Iterative 3D classification and alignment runs were executed until no further changes were observed in the final density maps. For experiments in which sCD4 or 17b were present, where the data appeared to be more heterogeneous, we used a more advanced collaborative alignment and clustering algorithm based on the concept of minimizing matrix rank and its convex surrogate, the nuclear norm [33], to derive the structures of the liganded complexes. Coordinates (PDB IDs: 3DNN, 3NGB, 3SE8, 2NY7 & 1GC1) were fitted into density maps using the software package UCSF Chimera [34]. A list of all complexes and coordinates used in tomographic studies is provided in Table 1.

**Table 1:** List of density maps and coordinates used for fitting.

Dataset	Crystal Structure Coordinates (PDB ID #)	Cryo-EM Method	Citation	Relevant Figure(s)
HIV-1 BaL	3DNN	Tomography	Liu, *et al*. 2008	1a, 1b
HIV-1 BaL+VRC01 IgG	3NGB	Tomography	This study	2e, 2f, 5, S5
HIV-1 BaL+VRC02 IgG	3NGB	Tomography	This study	S4e, S4f, S5
HIV-1 BaL+VRC03 IgG	3SE8	Tomography	This study	2g, 2h, S5
HIV-1 BaL+b12 Fab	2NY7	Tomography	Liu, *et al*. 2008	5
HIV-1 BaL+sCD4	1GC1 (without 17b Fab)	Tomography	This study	1c, 1d
HIV-1 BaL+17b IgG	1GC1 (without CD4)	Tomography	This study	1e, 1f
HIV-1 BaL+17b Fab	1GC1 (without CD4); 1AIK	Single Particle	This study	9a, 10, 11
HIV-1 BaL+17b Fab+sCD4	1GC1	Tomography	Liu, *et al*. 2008	1g, 1h
HIV-1 BaL+VRC02 Fab	–	Tomography	This study	S4e, S4f
HIV-1 BaL+VRC01 IgG+17b IgG	3NGB; 1GC1 (without CD4)	Tomography	This study	7b, 7c

### Single particle data analysis

A total of 3,299 micrographs, with defocus values spanning a range from 1–10 µm, were collected, and of these, a subset of 1,639 non-astigmatic, contamination-free, and artifact-free micrographs were selected based on visual inspection of each binned image, its 2D-Fourier transform and the output of two independent whole image CTF determinations using the programs tomoctffind [35] and ctffind3 [36]. Single particle analysis was carried out using EMAN2 [31], [37]. Micrographs were band-pass filtered and subject to semi-automated particle selection using a box width of 168 Å. A few representative particles were manually selected from the first micrograph and seeded to initiate the automated particle picking routine. Automatically picked boxes were edited manually to remove entries that could be clearly discarded as junk, and the procedure was repeated for an initial set of 193 micrographs to yield 20,541 2D projection images of individual Env complexes. The number of selected boxes per micrograph varied from 21 to 257. Particles were extracted with a box size of 288×288 pixels, CTF parameters for each micrograph were determined from individual particle stacks using the program e2ctf.py in the EMAN2 suite [37], and the results were compared with the previously obtained values as criterion for inclusion in the dataset (defocus range was 1.952 to 5.012 for the final set of particles used). A set of 19,080 particles was then subject to eight iterations of 2D classification. Inspection of the resulting 128 class averages confirmed the heterogeneous nature of the sample, which separated into unliganded (13,337 particles) and 17b-complexed (5,451 particles) images. Separate sets of eight initial models for soluble Env and the complex with 17b Fab were built from the segregated particle sets. Initial models were selected based on visual similarity with lower resolution models of the complexes obtained by cryo-electron tomography and subvolume averaging [23]. Selected models were subject to five independent iterations of refinement against the corresponding particle datasets. A second set of 102,700 individual projection images was then extracted from the entire micrograph data set, CTF-corrected as above for the smaller data set, and subjected to five iterations of refinement using the two reference models generated in the previous step. The re-projections from this final model are presented in Figure S6.

## Results

### Open quaternary conformation generated by CD4 or 17b binding

**Figure 1:**
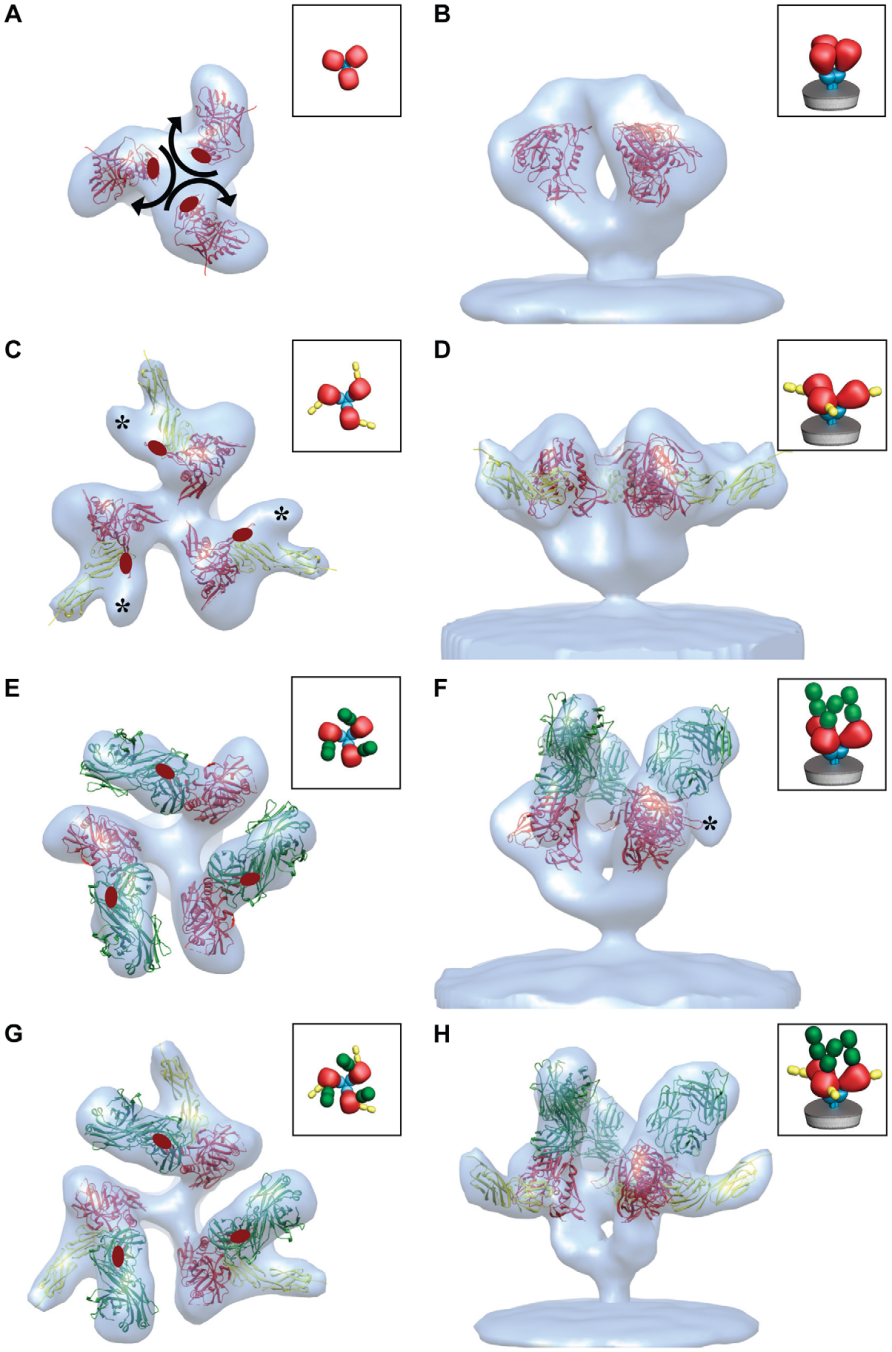
Binding to sCD4 or 17b is sufficient for formation of the open quaternary Env conformation. (a, b) Density map and molecular architecture for native trimeric Env from native HIV-1 BaL shown in top (a, from the apex) and side (b) views [21]. The locations of the stumps of the V1V2 loop are shown by the red ovals in (a) and subsequent panels with top views, and the black arrows indicate the rotation of each gp120 monomer that must occur when the trimer transitions from closed to open conformations. (c–h) Top and side views of the sCD4-bound (c, d) 17b-bound (e, f) and sCD4/17b bound [21] (g, h) Env density maps. Maps in all panels were fitted with subsets of the X-ray coordinates from the gp120-sCD4/17b complex (PDB ID:1GC1). The black asterisks (c, f) denote the location of the V1V2 loop. The insets schematically depict trimer conformation and bound ligands highlighting gp120 (red), gp41 (cyan), sCD4 (yellow) and 17b (green).

Biochemical analyses have established that sCD4 binding to monomeric gp120 induces local changes in conformation [38], [39], [40], [41]. In previous cryo-electron tomographic studies [21], we determined that native trimeric Env undergoes large quaternary changes upon simultaneous binding of sCD4 and the co-receptor mimic, 17b [42], to gp120 on intact HIV-1 BaL virions. Each gp120 protomer is rotated away from the central 3-fold axis to create an opening at the apex of the trimer. By fitting three copies of the atomic coordinates of the monomeric gp120/sCD4/17b complex into the density map, we derived a molecular model for this complex [21]. To test whether the binding of sCD4 alone or 17b alone results in measurable conformational changes, we carried out cryo-electron tomography (see Figures S1, S2, S3 for more information) of HIV-1 BaL virions after incubating them individually with soluble sCD4 or 17b. Envelope glycoprotein spikes are clearly visible on the membrane surface for viruses incubated with either 17b or sCD4 (Figure S1c, S1d). Analysis of density maps derived from these complexes of trimeric Env shows that binding of sCD4 alone, or 17b alone, results in a quaternary structural change in trimeric Env density similar to that observed when both sCD4 and 17b are bound (Figure 1). The quaternary structural change observed with 17b alone is surprising because HIV-1 BaL is a CD4-dependent strain and not expected to bind co-receptor molecules or a co-receptor mimic in the absence of CD4. Since no crystallographic structures have been previously reported for the complex of monomeric gp120 with sCD4 alone or with 17b Fab, we fitted the relevant subsets of the gp120/sCD4/17b molecular coordinates into the density maps to obtain a molecular interpretation of the sCD4 and 17b-bound states. In each case, there is a rotation of each gp120 protomer that repositions the V1V2 loops relative to that in the unliganded state (Figures 1a, 1b), as depicted by the movement of the stumps of the V1V2 loops in the fitted coordinates. The fits reveal that the gp120 portions of the sCD4-bound (Figures 1c, 1d) or 17b-bound trimers (Figures 1e, 1f) display molecular orientations in these separate binary complexes similar to the gp120 portion in the sCD4-17b-bound ternary complex (Figures 1g, 1h). This result establishes that 17b, which is referred to as a CD4i (CD4-“induced”) antibody because CD4 binding enhances its binding to Env, can bind and generate an open quaternary conformation of the Env spike in both the presence and absence of sCD4. We conclude that, while CD4 binding is sufficient for formation of the open quaternary Env conformation in HIV-1 BaL and in soluble Env trimers, binding of an antibody targeted to the co-receptor binding site can also generate a similar conformational change (at ∼20 Å resolution), in the absence of CD4. Structural models for trimeric Env in which the V1V2 loops are located at the base of the Env trimer [13] or mechanisms for CD4-mediated structural changes that invoke major rearrangements in the inner core of gp120 [43] are not consistent with the results we present here. Thus, while the binding efficiency of CD4i antibodies, such as 17b, is increased by pre-binding of CD4 [5], 17b binding to HIV-1 BaL Env is able to trigger formation of the open quaternary Env conformation even in the absence of CD4.

### Complex formed by trimeric HIV-1 Env with VRC01 and related antibodies

**Figure 2:**
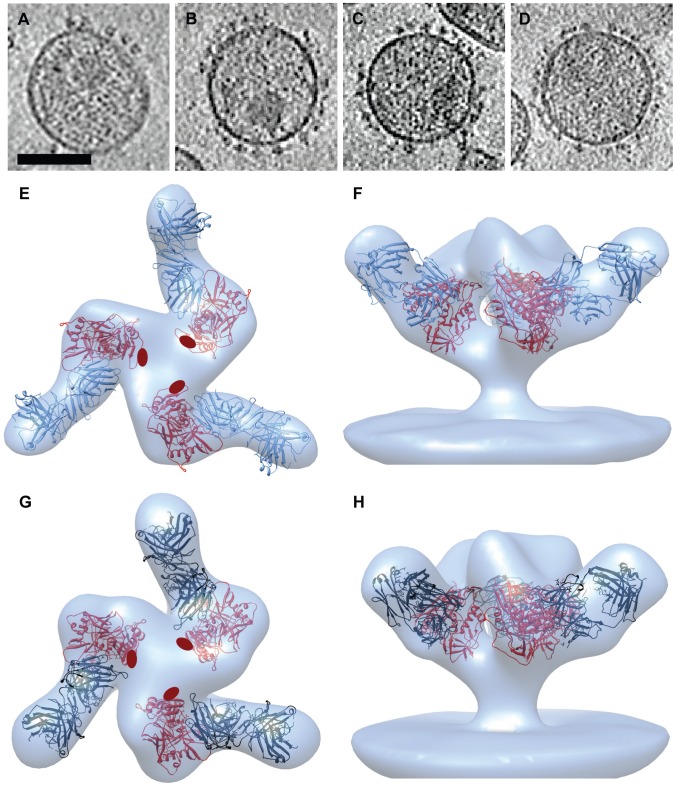
Structure of trimeric HIV-1 BaL Env bound to VRC01 and VRC03 antibodies. (a–d) Slices through reconstructed cryo-electron tomograms of HIV-1 BaL in the unliganded state (a) or complexed with VRC01 (b), VRC02 (c) or VRC03 antibodies (d). Peripheral Env glycoprotein spikes are visible on the viral membrane. Scale bar is 100 nm. (e, f) Top and side views, respectively, of the VRC01-bound HIV-1 BaL Env density map fitted with X-ray coordinates of the gp120-VRC01 Fab complex (PDB ID:3NGB). (g, h) Top and side views, respectively, of the VRC03-bound Env density map fitted with X-ray coordinates for gp120-VRC03 Fab (PDB ID:3SE8). Coordinates show gp120 (red), VRC01 (blue) and VRC03 (black). In panels (e) and (g), the locations of the stumps of the V1V2 loop are shown by the red ovals.

The binding interfaces between gp120 and sCD4 and between gp120 and the broadly neutralizing antibody VRC01 are very similar, and share many of the same residues on gp120 [16]. Despite these similarities, the functional consequences of trimeric Env binding to VRC01 or sCD4 are profoundly different [25]. We therefore carried out cryo-electron tomographic experiments to determine if the quaternary structure of VRC01-bound Env is different from the open Env structure observed upon sCD4 binding. Starting from cryo-electron tomograms of VRC01-, VRC02- or VRC03-bound viruses (Figures 2a–2d), we then determined the 3D structures of these trimeric Env complexes by classification and 3D averaging. Density maps of trimeric Env bound to VRC01 show the expected additional density from the bound antibodies, which project upwards from the apex of the spike towards the target cell membrane (Figures 2e, 2f). By fitting three copies of the X-ray structure of monomeric gp120 with VRC01 Fab (PDB ID: 3NGB) into the map, we obtained a molecular model for the VRC01-bound Env trimer. Similar tomographic experiments carried out with VRC03-bound viruses resulted in a density map for the complex with Env. We fitted three copies of the gp120-VRC03 coordinates (PDB ID:3SE8) into the density map of trimeric Env bound to VRC03 to derive a molecular model for this complex on the surface of the virus (Figures 2g, 2h). The quaternary structures of trimeric gp120 complexed with VRC01 and VRC03 are similar, with the bound antibodies projecting at an angle of ∼30° from the apex of the spike towards the direction of the target cell membrane.

X-ray structures for the gp120-VRC02 complex have not been reported, but the density maps of trimeric Env complexed to VRC02 (Figure S4) are very similar to those obtained with VRC01 and VRC03, consistent with the expectation that all three antibodies recognize similar binding sites on gp120 [25]. The density maps for the Env-VRC02 complex were obtained using whole IgG, as in the case of the maps with VRC01, VRC03 and 17b, but only the density corresponding to one Fab fragment is visible on each gp120 monomer in each of these density maps. Because of the inherent flexibility of the hinge regions in antibody molecules, portions (i.e. the other Fab and Fc segments) not in direct contact with gp120 have random orientations relative to Env, and the density from these portions is therefore smeared out in the overall density map. We used the complex of trimeric Env with purified Fab fragments to validate this point. Direct comparison of density maps obtained for Fab-bound and IgG-bound trimeric Env complexes (Figures S4a–S4d) are similar and superimposable (Figures S4e, S4f). In addition, both of these VRC02-bound complexes are similar to the complexes formed by VRC01 and VRC03 as shown by the superposition of all three IgG-bound density maps (Figure S5).

**Figure 3:**
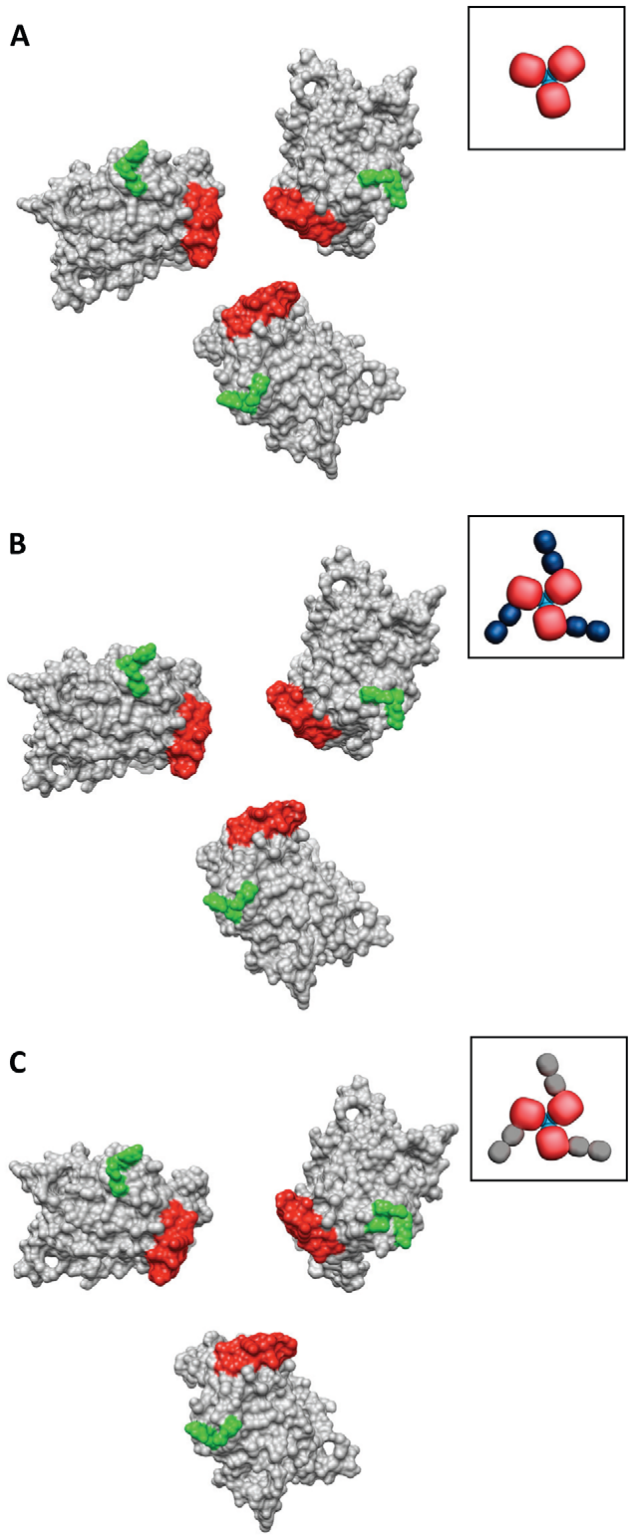
Similar quaternary conformations of native unliganded HIV-1 Env and Env bound to VRC01 or VRC03. (a–c) The gp120 portion of the gp120-VRC01 X-ray coordinates (PBD ID:3NGB) is shown after alignment to gp120 fitted to either the unliganded (a), VRC01-bound (b) or VRC03-bound (c) Env density maps to show the similarity in gp120 conformation in each of these states. The orientation of the stumps of the V1V2 (red) and V3 (green) loops on the gp120 surface provides a visual marker for gp120 conformation. Cartoon representations of the gp120 coordinates (unliganded or ligand-bound) are shown next to each set of coordinates, with gp120 (red), gp41 (cyan), VRC01 (blue) and VRC03 (dark grey).

**Figure 4:**
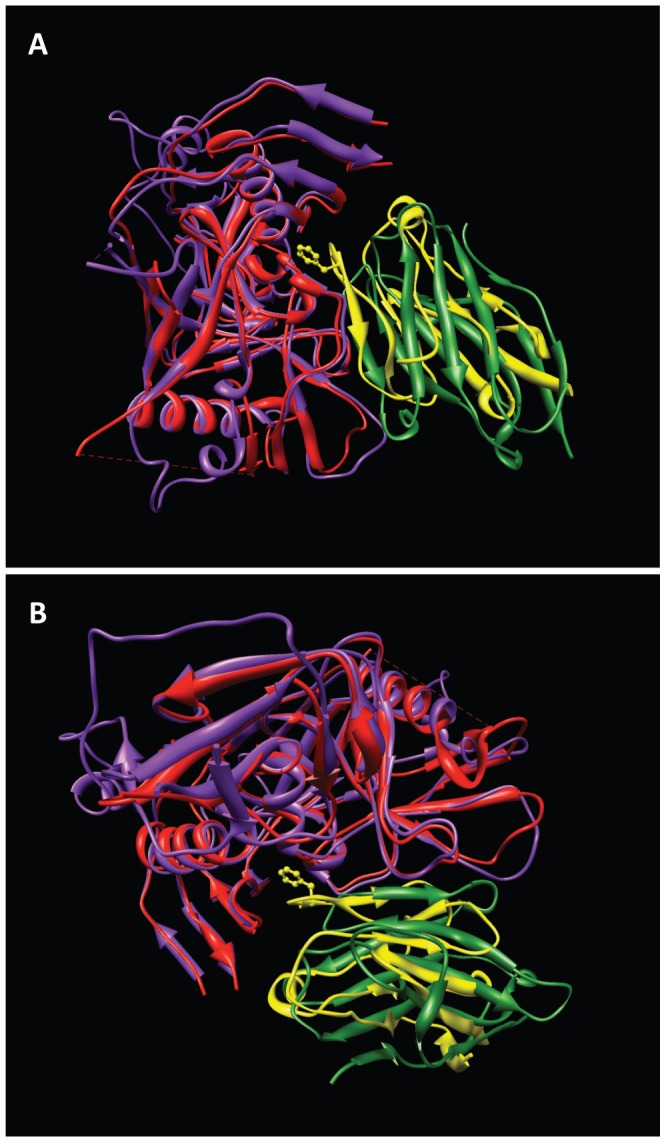
Similarity in structure of regions of sCD4 and VRC01 that contact gp120. (a, b) Two orthogonal views of the superposition of gp120 polypeptides derived from the complex with sCD4 and 17b (PDB ID: 1GC1) and the complex with VRC01 (PDB ID: 3NGB). For clarity of visualization, only those regions of VRC01 and sCD4 that are in close proximity to gp120 are shown. The gp120 chain and sCD4 chains from the 1GC1 structure are shown in red, and yellow, respectively, while the gp120 and VRC01 chains from the 3NGB structure are shown in magenta and green, respectively.

The molecular structures of trimeric Env bound to VRC01 or VRC03 antibodies (Figure 3) is very close to that obtained previously for unliganded trimeric Env [21] (PDB ID: 3DNN). Thus, despite the remarkable similarities in sCD4 and VRC01 interaction with gp120 (Figure 4), VRC01 (and also VRC02, VRC03) binding retains the closed, unliganded trimer conformation, while sCD4 binding results in transition of Env to the open trimer conformation. Consistent with these dramatic differences in the structural consequences of CD4 or VRC01 binding, spike shedding, which is known to occur in the presence of CD4 [44], [45], [46] is not induced by VRC01 [47].

### Comparative analysis of CD4-binding site ligands

**Figure 5:**
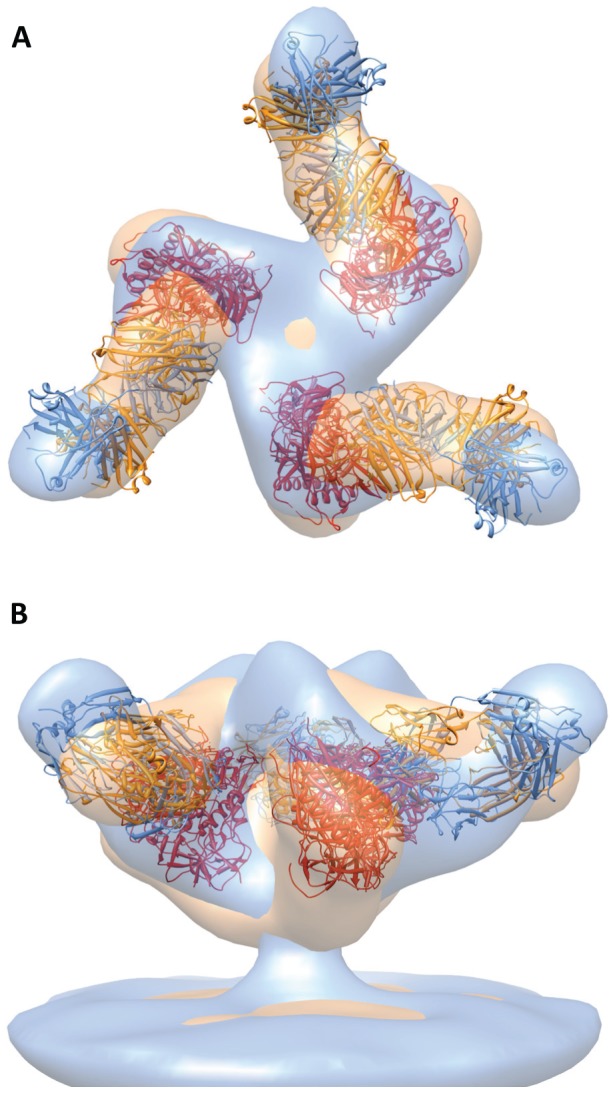
Comparison of structures of trimeric Env complexed to either VRC01 or b12. (a, b) Top and side views, respectively, showing the superposition of VRC01-bound (blue) or b12-bound (orange) Env density maps fitted with the corresponding gp120-VRC01 (PDB ID:3NGB) or gp120-b12 (PDB ID:2NY7) coordinates. The gp120 components of the coordinates are shown in red for both complexes, while the VRC01 and b12 Fab fragments are shown in blue and orange, respectively.

**Figure 6:**
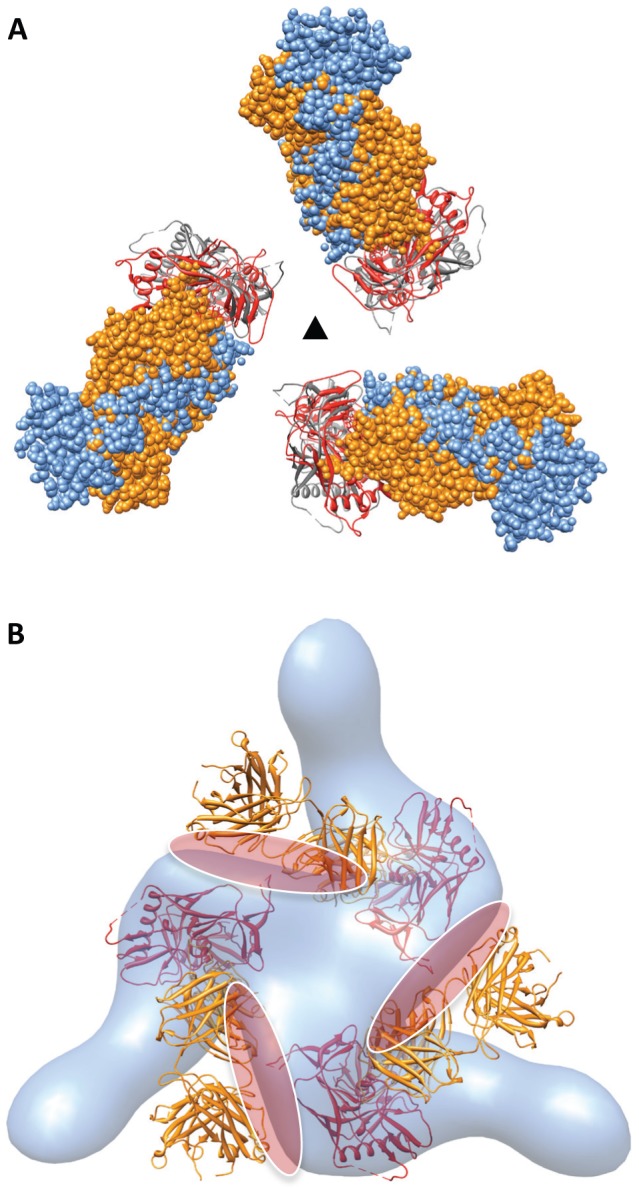
Binding of VRC01 retains Env in the closed state, but b12 binding requires an in-plane rotation of gp120. (a) Top view of the molecular coordinates for gp120 bound to VRC01 (gp120 in red, VRC01 in blue, PDB ID: 3NGB) or bound to b12 (gp120 in grey, b12 in orange, PDB ID: 2NY7). Env binding to b12 results in a small in-plane rotation of gp120, while VRC01 binding retains the unliganded gp120 conformation. (b) The gp120 portion of the b12-bound complex was aligned to the gp120 portion of the VRC01-bound complex to model b12 binding in the VRC01-bound, closed, conformation. Coordinates of the gp120-b12 complex are shown with the VRC01-bound Env density map. The projected steric overlap between b12 Fab and neighboring gp120 protomers in the closed state is highlighted for visualization purposes with the red elliptical patches. Coordinates for gp120, b12 and VRC01 are in red, orange and blue, respectively.

Comparison of the density map and fitted coordinates of the gp120-VRC01 trimer with that of the gp120-b12 trimer provides a new insight into the different conformations observed when different neutralizing antibodies are bound to Env (Figure 5). As viewed from the apex of the spike, the direction of the Fab densities extending outward from the spike is roughly similar for both antibodies (Figure 5a). However, a side view reveals that the b12 Fab fragment is attached to gp120 slightly higher relative to the viral membrane as compared to VRC01, and is oriented roughly parallel to the membrane in contrast to VRC01, which is oriented upwards (Figure 5b). Despite general similarities in their overall architectures, there are dramatic differences in the quaternary structure of trimeric gp120 between these gp120-antibody complexes. Inspection of the fits shows that, in order to accommodate b12, each gp120 protomer in the trimer is rotated outward by an in-plane rotation of ∼20° relative to the orientations in the VRC01 complex (Figure 6a). If b12 binding were to occur without any changes in conformation from that observed in the complex with VRC01, the orientation of the bound antibody would be sterically restricted because of contacts with the rest of the trimer (Figure 6b). These results elucidate a key difference in the binding between these neutralizing antibodies to trimeric Env on the native viral surface: while VRC01, 02 and 03 are capable of binding trimeric Env in its native state without significant rearrangement in the quaternary conformation, an outward rotation of each gp120 protomer by ∼20° is essential for b12 binding. Some level of conformational flexibility in the binding site on gp120 may be required to accommodate initial contact with b12; subsequent rearrangement by an “induced-fit” type of mechanism [48] could then result in the partially open quaternary state observed in the density maps. The requirement for a rearrangement of the quaternary structure may limit neutralization ability of b12 to only strains in which packing constraints in trimeric Env are loose enough to allow these gp120 rotations. These findings explain the results from biochemical analyses which have shown that b12 binding results in a greater conformational change in gp120 as compared to that observed with VRC01 [47], that b12 and 17b cannot simultaneously bind gp120 [25] and that mutations in HIV-1 gp120 can be identified which result in escape from neutralization by b12, but not by VRC01 [49].

### VRC01 binding locks Env in a closed state

**Figure 7:**
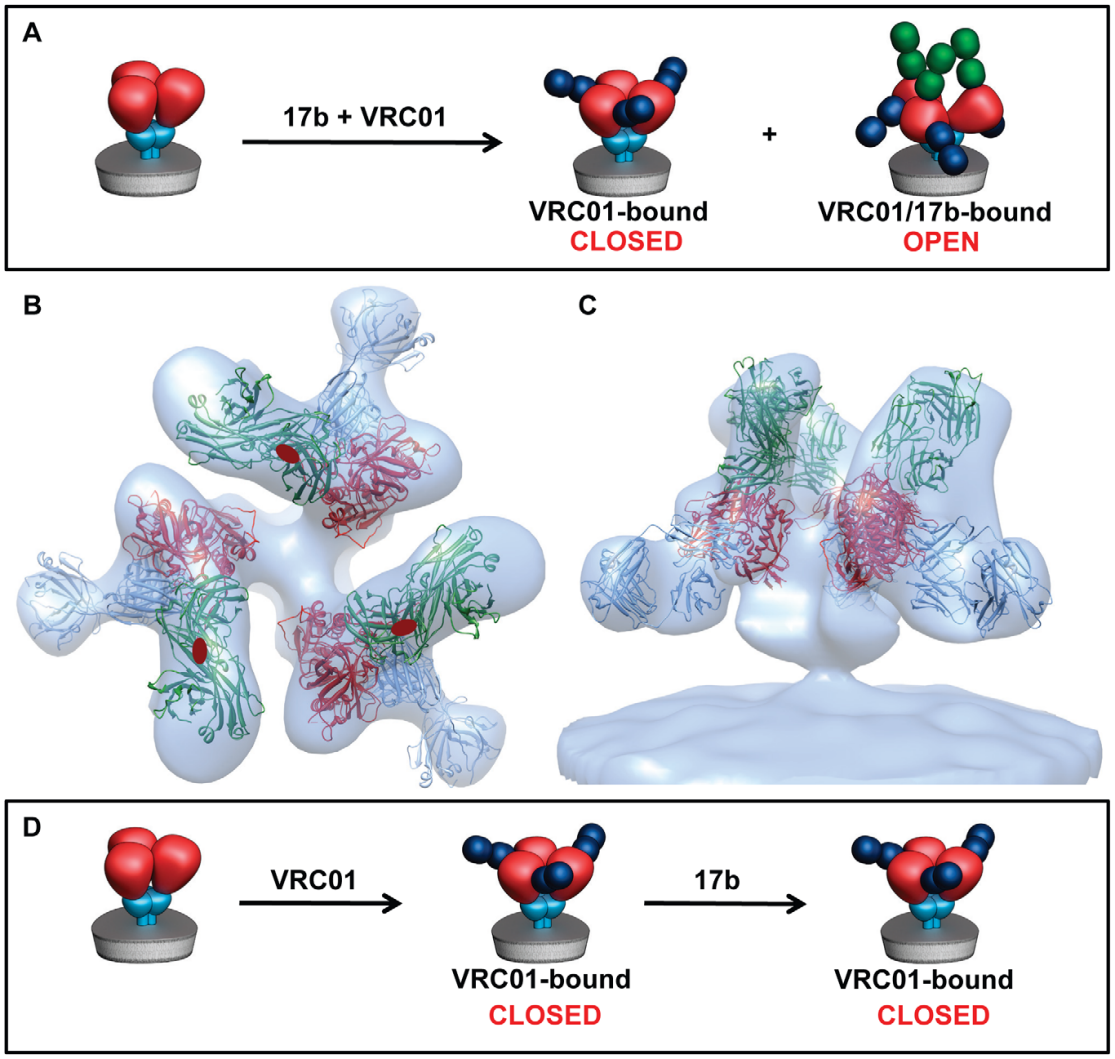
Conformational states of trimeric Env in the presence of VRC01 and 17b. (a) Schematic representation of the Env maps obtained after incubation of HIV-1 BaL with 17b and VRC01. (b, c) Top and side views, respectively, of the VRC01/17b-bound Env density map. X-ray coordinates for the gp120-VRC01 complex (PDB ID:3NGB) were fitted into the map and subsequently aligned to the gp120-CD4/17b (PDB ID:1GC1) complex. (d) Schematic illustration of the finding that when VRC01 is pre-bound to HIV-1 BaL, all detectable Env is in a closed VRC01-bound state, and this is not altered upon subsequent addition of 17b.

Biochemical experiments have shown that binding of either sCD4 or VRC01 can enhance the binding of 17b to monomeric gp120 [25], [40]. This contrasts with the differing effects of sCD4 and VRC01 on the quaternary conformation of trimeric Env (Figures 1, 2). To test whether VRC01 binding influences 17b binding in the context of native trimeric Env, we performed experiments in which 17b and VRC01 were both added to HIV-1 BaL (Figure 7). 3D classification of trimeric Env subpopulations revealed the presence of two distinct antibody-bound populations. In the first, only VRC01 was bound, and Env was in the closed conformation. In the second, both 17b and VRC01 were bound, and Env had an open conformation (Figures 7b, 7c). Importantly, in these latter complexes, the bound VRC01 is angled downwards towards the viral membrane, as expected from the out-of-plane rotation of gp120 that occurs with transition from the closed to the open conformation. No closed, 17b-bound state was detected. These experiments suggest that envelope glycoprotein spikes that were bound by VRC01 first retain the closed conformation and block 17b binding, while those that were bound by 17b first and transitioned to the open state allow subsequent binding of VRC01. To test this hypothesis, we pre-incubated virus with VRC01 for an hour before adding 17b, and determined the distribution of antibody-bound Env complexes. Under these conditions, all detectable Env displayed the closed, VRC01-bound conformation with no evident 17b or VRC01/17b-bound populations (Figure 7d). We conclude that there are structural constraints that prevent binding of 17b in the VRC01-bound closed conformation; these constraints are not present in the closed, unliganded state, which can bind 17b and transition to the open state. The potent neutralization ability of VRC01 may therefore be attributed to its ability to block the CD4- or co-receptor- triggered transition of Env to the open state.

### Soluble trimeric Env forms a novel “activated” intermediate upon binding to a coreceptor mimic

The structural studies we have presented show that binding of CD4 and/or 17b, a co-receptor mimic, can lead to a large quaternary change in the arrangement of gp120 in trimeric Env. To provide further insight into epitopes exposed by activation of Env, we used single particle cryo-electron microscopic methods to determine structural changes that occur upon 17b binding at resolutions higher than that achieved by tomography. For this purpose, we used SOSIP Env trimers from the Clade A strain KNH1144 [50]. These trimers are soluble, proteolytically cleaved trimers that are stabilized by the presence of an engineered intermolecular disulfide bond between gp120 and gp41 (SOS), combined with a single residue change, I559P, within gp41 [51]. Importantly, we have shown previously that these trimers, which contain the complete ectodomain of trimeric Env, display the same 17b-induced change in quaternary structure we report here for native trimeric Env [23].

**Figure 8:**
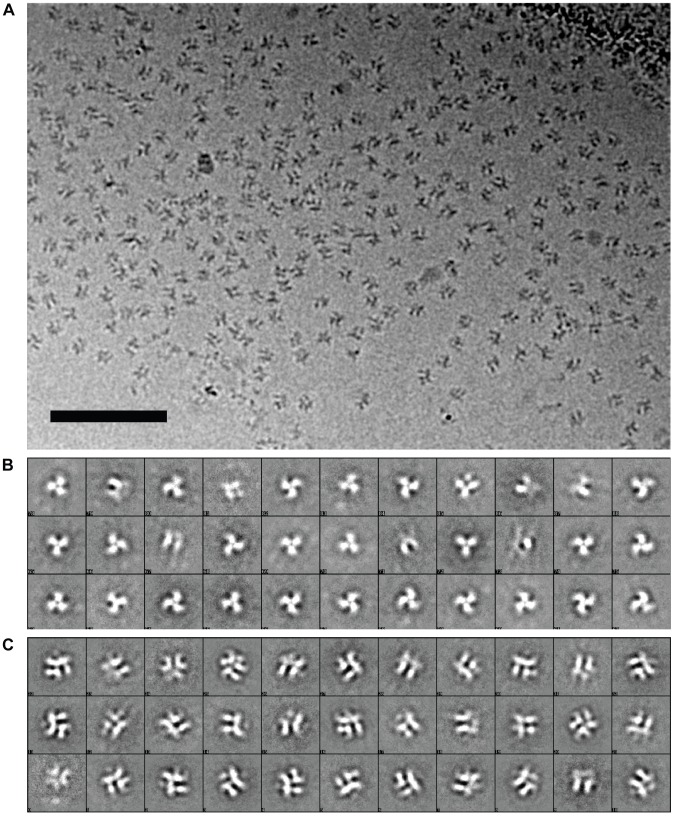
Cryo-electron microscopy of the complex formed between soluble KNH1144 trimeric Env and 17b Fab fragments. (a) Projection image recorded on a Titan Krios electron microscope operated at 80 kV using a 4K×4K CCD camera. Scale bar is 50 nm. (b, c) Representative examples of initial 2D class averages separating unbound or partially occupied complexes (b) from those that appear to have full occupancy of 17b (c).

Cryo-electron microscopic images of soluble trimeric Env incubated with 17b Fab fragments show the presence of well-separated molecular complexes (Figure 8a). Classification of the projection images allows separation of the unliganded trimeric gp140 and other partially bound species (Figure 8b) from 17b-bound complexes (Figure 8c). Initial model building allowed visualization of the 3D structure and verification that the re-projections matched the projection image classes obtained from the raw data (Figure S6a). Progressive refinement (see Methods for details) results in progressively higher amount of detail emerging in the map, as evidenced by comparing the re-projections of the final map (Figure S6b) as compared with the initial map (Figure S6a).

**Figure 9:**
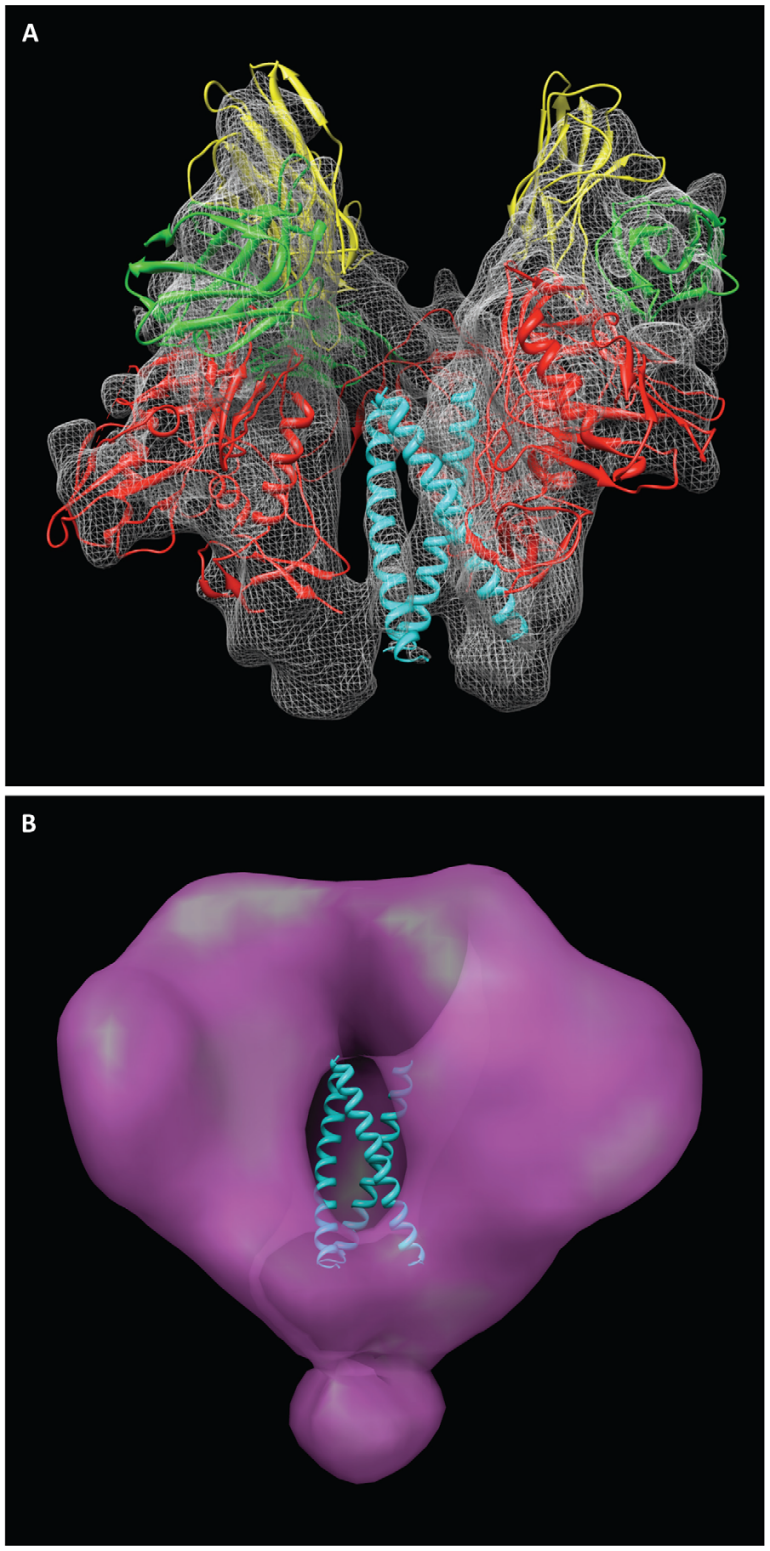
Structure of the open conformation of trimeric Env at sub-nanometer resolution. (a) Side view of the structure of trimeric Env bound to 17b Fab. The map was fitted with three copies of the X-ray structures for the gp120-17b portion of the 1GC1 coordinates with gp120 (red) and 17b Fv fragments (light chain: yellow, heavy chain: green). One copy of the gp41 N-terminal helix (cyan) of 1AIK coordinates (N34) was fitted individually into each of the three densities, which occupy the central region of the spike that is essentially a cavity in the unliganded state. (b) Side view of the density map from unliganded native trimeric Env [21], [22], [23], [24], with the three gp41 N-terminal helices (cyan) superposed to show that in the open conformation, they occupy the solvent filled cavity in the density map of the unliganded state.

The structure of the soluble Env-17b Fab complex at ∼9 Å resolution shows the extensive rearrangement in the N-terminal region of gp41 as Env transitions from the closed to open conformation (Figure 9a). From fitting three copies of the gp120 and 17b subset of 1GC1 coordinates of the gp120-sCD4-17b complex [12] we obtained a molecular model for the gp120 and 17b components of this complex. The densities for the three gp120 protomers as well as the Fv portion of 17b are well-resolved and match the coordinate fits for the ternary complex. At the early stages of the refinement, density for the entire Fab is visible, as in the case of the maps derived by tomography of 17b-bound trimeric Env complexes [21], [23]. With progressive refinement and improved alignment of the gp41 and gp120 components of the images, the alignment of the Fv portion improves at the expense of the rest of the Fab because of the flexibility of the hinge regions at the center of the Fab fragment.

A prominent feature of the density map is the appearance of three long densities, which we attribute to α-helical segments, resolved at the center of the complex (Figure 9a). These helices span a region that is essentially a solvent-filled cavity in the unliganded state (Figure 9b). These helices were not resolved in the lower resolution tomographic density maps of sCD4/17b-bound native Env trimers [21], 17b-bound native trimers (Figures 1e, 1f) or in 17b-bound SOSIP gp140 trimers [23], where they appear as unresolved blobs of density at the center of the spike (see Figure 1h). Since we know that this additional density in the middle can only come from gp41, there are only two possible assignments for the long rods of density: either they represent the N-terminal gp41 helices or they represent the C-terminal gp41 helices. If the density is assigned to the C-terminal helix, it would result in placement of the free end of the helix corresponding to the MPER (membrane proximal extended region) at the apex of the spike facing the target cell membrane. This is, however, not a plausible assignment, as the MPER peptide is the region immediately adjacent to the transmembrane region of gp41, and must reside at the base of the spike. We conclude that the rods of density in the middle must therefore arise from the N-terminal helix. There is no ambiguity about the orientation of the N-terminal helix in the structure because we know from the sequence of gp41 that the fusion peptide resides at the free end of the N-terminal helix, and that the C-terminal end is connected to the rest of the polypeptide.

**Figure 10:**
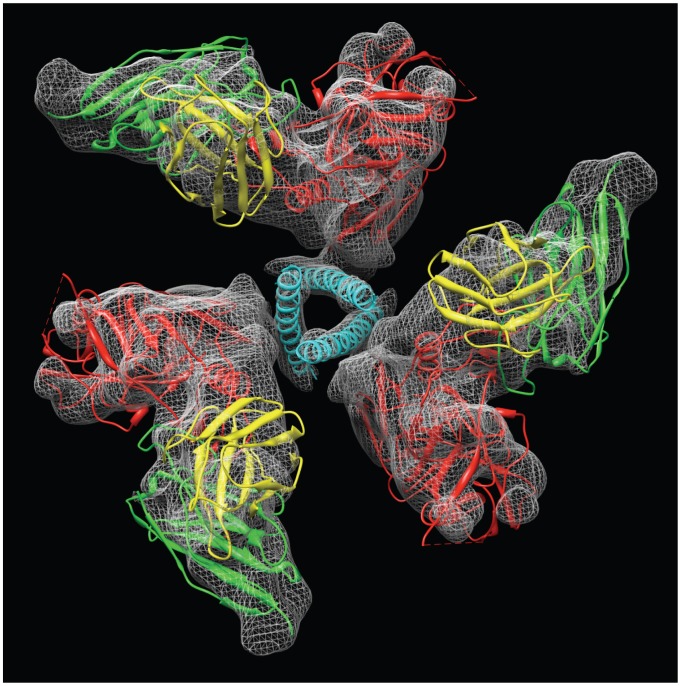
View of the central gp41 helices in the open conformation of trimeric Env bound to 17b Fab. The map was fitted with three copies of the X-ray structures for the gp120-17b portion of the 1GC1 coordinates with color scheme as in Figure 9.

**Figure 11:**
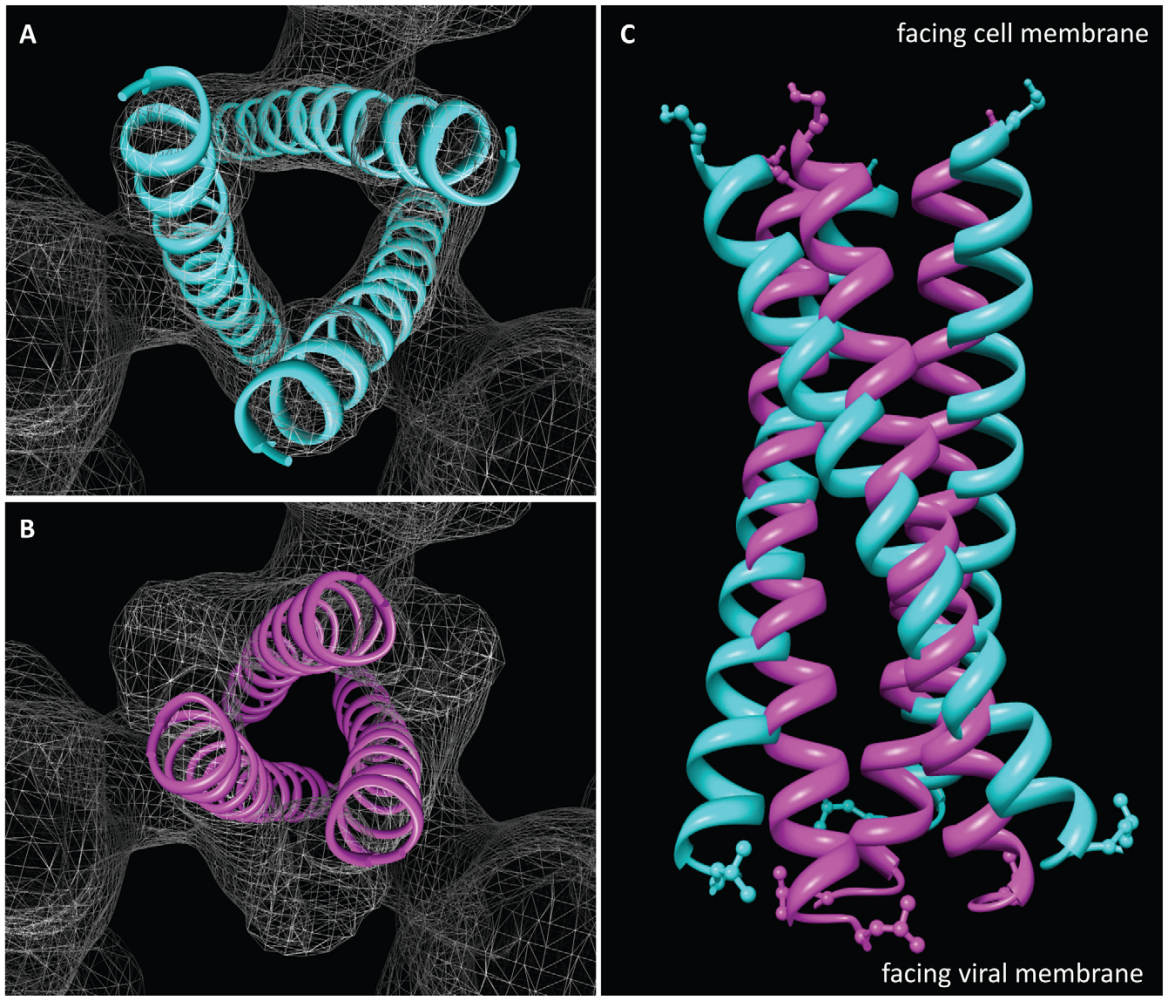
Mechanism of activation of trimeric Env. (a) Top view of the locations of the three N-terminal helices in the open conformation (as in Figure 10). (b) Top view of the locations of the same helices in the crystal structure (1AIK) of the six-helix bundle formed by the three N- and three C-terminal helices, illustrating the more compact packing in the post-fusion state. (c) Superposition of the arrangement of the three N-terminal helices in the open quaternary Env conformation (cyan) derived from cryo-electron microscopy of soluble trimeric Env with that in the post-fusion conformation (magenta) derived by X-ray crystallography of the six-helix bundle.

By fitting coordinates for the N-terminal gp41 helix (N34; PDB ID: 1AIK) into the density map, we arrive at an assignment in which the fusion peptide is at the free end facing the target cell membrane and is connected to the rest of the polypeptide at the bottom on the side of the viral membrane. The N-terminal end of this helix is located ∼15 Å–20 Å below the top of the spike and forms an arrangement of three tilted helices arranged around the central 3-fold axis of the trimer (Figure 10). The entire length of the 34-amino acid long peptide fits into the density, and we presume that the less-ordered 26-amino acid fusion peptide of gp41 or the polypeptide segment connecting the fusion peptide to the N-terminal helix are not visible in our map. The fits to the density map show that the three N-terminal helices are held apart by close interactions with the rest of the Env ectodomain, and are not in the closely packed coiled-coil structure observed in the post-fusion state [10], [18], [19]. The looser packing of the three gp41 N-terminal helices likely arises from the additional disulfide bind present in SOSIP gp140 trimers between the gp120 and gp41 polypeptides. Comparison of the packing arrangement of the gp41 N-terminal helices in the open quaternary conformation (Figure 11a) with that determined by X-ray crystallography and NMR spectroscopy for the post-fusion state (Figure 11b) reveals the structural change that must occur during this transition (Figure 11c). The transition from the open quaternary conformation to the post-fusion state requires a change in angle by ∼15° of each of the three N-terminal helices. In addition, the helices become more compactly packed as a result of their interaction with each other and the C-terminal portion of gp41.

Previous models for the mechanism of HIV entry have postulated that activation of Env by CD4 and co-receptor binding should lead to formation of a transient “pre-hairpin” intermediate [2], [19], [52], in which the N-terminal helices of gp41 become exposed and accessible to binding by entry inhibitors [53]. No 3D structure is available for this intermediate, which by definition is a transient intermediate that is formed when the exposed fusion peptide makes contact with the target cell membrane. There is a large body of evidence indicating that the pre-hairpin intermediate contains a tightly packed three-stranded coiled-coil core and that this state is inhibited by drugs such as enfuvirtide [7]. In the structure we describe here, the N-terminal helices are not closely packed, and there is no target membrane for insertion of the fusion peptide. We therefore favor the view that we have captured an activated intermediate whose formation precedes that of the pre-hairpin intermediate. The structure of our activated intermediate nevertheless provides an explanation for many of the biochemical properties attributed to the pre-hairpin intermediate. Thus, the absence of close packing of the N-terminal gp41 helices in the pre-fusion, open state, and their close association with the rest of Env explains the biochemical observation that they are unable to form stable trimers on their own unless they are artificially stabilized [54]. Further, in our structure, the gp41 N-terminal helices show a larger opening at the side of the target cell and greater burial on the side of the viral membrane. This gp41 arrangement accounts for the large differences observed in the accessibility of larger peptides to the different ends of the gp41 N-terminal helix [8]. This “steric restriction” of access to gp41 by the asymmetric environment of the N-terminal helix may also explain why pre-hairpin intermediate reactive antibodies such as D5 [6], which bind the viral-membrane side of the gp41 N-terminal helix, are much more effective as scFv fragments than as whole antibodies [8]. Whether the N-terminal helix is accessible to gp41-reactive antibodies such as 8066 [55], D5 [56] or HK20 [57] in the intermediate stage we have trapped, and whether structural changes occur in gp41 and or gp120 as a result of binding are important questions that can now be addressed. It is also possible that some of the properties attributed to the pre-hairpin intermediate may be attributable to the activated intermediate, and this also remains to be tested.

**Figure 12:**
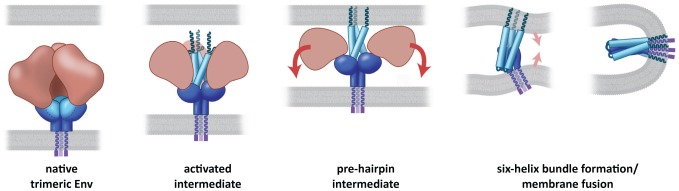
Model for the mechanism of Env activation. The CD4- or co-receptor-triggered activation of the Env spike forms an activated intermediate in which the N-terminal gp41 helices are vulnerable to neutralizing ligands. The pre-hairpin intermediate is formed upon insertion of the fusion peptide into the target cell membrane and dissociation of gp120, leading subsequently to formation of the six-helix bundle state and subsequent fusion between viral and target cell membranes.

From our results, we propose that the open quaternary conformation of trimeric Env is an activated intermediate that is a precursor to the pre-hairpin intermediate (Figure 12). In the activated intermediate, the N-terminal helix trimer is not physically separated from the rest of the Env trimer, as it is in schematic models for the pre-hairpin intermediate [2], [52]. Instead, we postulate that the pre-hairpin intermediate is formed by release of the constraints that hold the three gp41 N-terminal helices apart. This event is likely to occur with dissociation of gp120 upon insertion of the fusion peptide into the target membrane. This transient pre-hairpin intermediate decays on a time scale of many minutes, with the N-terminal helices undergoing further rearrangement to be part of a six-helix bundle that is composed of three inner (N-terminal) and three outer (C-terminal) helices. We anticipate that both the activated state (where gp120 has not yet dissociated), and the pre-hairpin intermediate state, where the N-terminal gp41 helices span the region between viral and cell membranes, are targets for antibodies such as D5. The structure we present here for the activated intermediate is such that the N-terminal helices are partially buried at the center of the open spike, and not in the extended state schematically envisioned for the pre-hairpin intermediate [2], but closer to the “committed” intermediate conformation suggested from entry kinetics studies [58]. Further, this assignment renders the arrangement of the fusion peptide in HIV entirely consistent with that seen for other viruses, such as influenza and Ebola, that use this mechanism of fusion to enter target cells.

## Discussion

In this work, we present a number of structures of trimeric HIV-1 Env and antibody complexes obtained by cryo-electron microscopy, including a 9 Å map of a novel, previously unknown activated intermediate that precedes formation of the pre-hairpin intermediate. The discovery (Figure 1) that binding of the co-receptor site on gp120 can induce the same quaternary conformational changes in trimeric Env as those that occur with CD4 binding is surprising. It is conceivable that the ability of 17b to induce a conformational change similar to that observed with CD4 is strain-specific, and that strains other than HIV-1 BaL may have a lesser propensity to undergo this 17b-induced change in the absence of added sCD4. In this context, we reported previously that, while soluble KNH1144 Env trimers were capable of displaying an open, 17b-bound conformation, similar trimers from JR-FL displayed the 17b-bound conformation only in the presence of bound sCD4 [23].

The differences in the quaternary structures of trimeric Env bound to either VRC01 or b12 were also unexpected; these findings provide a molecular basis for understanding the differences in neutralization efficiency of these two antibodies. A CD4 mimic may be expected to induce similar structural changes as sCD4 itself. However, we show now that this is not the case, based on determination of the quaternary structures of native trimeric Env bound to VRC01, VRC02 and VRC03 antibodies. Thus, despite the remarkable similarities in the interactions of sCD4 and VRC01 with gp120 (Figure 4), VRC01 (and also VRC02, VRC03) binding retains the unliganded (closed) trimer conformation, while sCD4 binding results in transition of Env to the open trimer conformation. Initial studies of VRC01 binding [25] suggested that VRC01 binding enhances binding of 17b to gp120 monomers. Our results now establish that this does not hold true for trimeric gp120, highlighting the importance of analyzing antibody binding to gp120 trimers in the context of the native virus. Furthermore, the evidence that 17b binding in intact viruses is actually blocked by VRC01 binding suggests a more general structural mechanism for neutralization by antibodies such as VRC01. Our results show that this “CD4 mimic” not only blocks CD4 binding by binding a similar region on Env, but that it actually prevents the opening of the spike necessary for exposure of the pre-hairpin intermediate, and by extension, infection.

Thus, a central finding from our comparative structural analyses is that binding of ligands, including antibodies with very similar footprints on gp120, as judged by crystallographic and mutagenesis studies, can have profoundly different outcomes for the conformation of trimeric gp120. Strains that are less susceptible to the type of conformational rearrangements in gp120 and gp41 that are required to accommodate b12 binding may therefore be less likely to be neutralized by antibodies such as b12, offering a possible explanation for the lower neutralization breadth of b12. The reason for poorer neutralization breadth of VRC03 relative to VRC01 may lie in lower affinity [25] for gp120, since there appear to be no significant differences either in the X-ray structures of their respective complexes with gp120 monomers [16] (PDB ID: 3ES8) or in the complexes formed with native trimeric Env (Figure 2). Whether the formation of the open quaternary state of Env is always sufficient to expose gp41 and lead to fusion between viral and target cell membranes remains an unanswered question. It also is unclear whether there are subtle variations in the quaternary structures, such as differential exposure of the gp41 fusion peptide in the open states populated by distinct ligand binding combinations.

Our description of the structure at ∼9 Å resolution suggests a new structural template for designing immunogens that can elicit antibodies targeting HIV at a vulnerable, pre-entry stage. Previous models for the mechanism of HIV entry have postulated that activation of Env by CD4 and co-receptor binding should lead to formation of a transient “pre-hairpin” intermediate, in which the N-terminal helices of gp41 become exposed and accessible to binding by entry inhibitors. No structure is available for this intermediate, but many biochemical experiments have been used to deduce its likely structural properties. In our work, we show that activation of Env by CD4/co-receptor binding leads to the formation of an activated intermediate where three N-terminal helices are nestled at the center of the complex, surrounded by trimeric gp120 that has moved outwards. It is likely that the presence of the additional disulfide bond and possible the Ile to Pro mutation in the SOSIP gp140 constructs stabilizes the activated intermediate. We further show that the N-terminal helices in this activated intermediate are not in the same compact structure that is observed for the post-fusion state. The differences are significant: the transition from the open quaternary conformation to the post-fusion state requires a change in angle by ∼15° of each of the three N-terminal helices. In addition, the helices become more compactly packed as a result of their interaction with each other and the C-terminal portion of gp41. Our discovery, therefore, suggests a new template for immunogens; antibodies elicited using this model could potentially have greater potency than those currently elicited using peptide mimics based on the compact post-fusion structure.

Conversion of the activated three-helix structure to the post-fusion six-helix bundle is a key event common to the entry mechanisms of many viruses, such as HIV, influenza, Ebola and Moloney murine leukemia virus [19]. Determination of the spatial arrangement of the gp41 N-terminal helices thus provides a structural template for vaccine design that is based on an experimentally observed intermediate state. This intermediate state could prove to be more immunogenic than templates derived from the structure of the post-fusion six-helix bundle state which occur at a late stage in the entry process [10]. The N-terminal helix of gp41 is one of the most conserved regions in HIV-1 Env. Because the open Env conformation occurs prior to membrane fusion and the N-terminal helices are more exposed than in the compact organization of the post-fusion state, immunogens based on this conformation are likely to elicit antibodies that could be effective in blocking entry across a broad spectrum of HIV-strains. Our observations are also consistent with the finding that, in HIV-1, dissociation of gp120 from gp41 exposes an epitope located in the loop between N- and C-terminal segments of gp41 [3], which is recognized by the murine monoclonal antibody KK20.

Overall, our findings are consistent with a model in which native trimeric HIV-1 is in a closed, but metastable conformation. Binding of ligands, such as sCD4, and co-receptor mimics, such as 17b, result in the formation of a dramatically different, activated intermediate conformation. In this conformation, gp120 and gp41 undergo coordinated structural changes, resulting in exposure of the gp41 fusion peptide through an opening at the apex of the spike. The structural analyses presented here demonstrate that different neutralizing antibodies block viral entry by distinct structural mechanisms. Antibodies such as VRC01 appear to neutralize HIV by binding the CD4 site and blocking activation of Env, thereby preventing the opening of the spike necessary for exposure of the fusogenic components of gp41 and subsequent viral entry. Antibodies such as b12 also appear to hold trimeric Env in the closed state, but with subtle rearrangements in the quaternary packing of gp120 in the trimer (Figures 5, 6). Therefore, the neutralization ability of b12 may be limited to strains in which packing constraints in trimeric Env are loose enough to allow these gp120 rotations, potentially explaining its lower neutralization efficacy compared to VRC01. In contrast to VRC01 and b12, 17b binding blocks entry by capturing a conformation in which the fusion peptide is physically prevented from contact with the target cell membrane by the bound antibody. In a recent study, Scheid et al [59] found that broadly neutralizing activity in serum from HIV-infected patients arose not from one highly potent neutralizing antibody, but from the aggregate activity of several antibody specificities. It is clear, therefore, that multiple antibodies can bind the same Env trimer simultaneously, and that the same antibody can be accommodated on the gp120 surface in very different quaternary states of trimeric Env. Effective strategies for HIV neutralization may thus require elicitation of a variety of antibodies, including those like VRC01 that lock gp120 and gp41 in the unliganded conformation, antibodies like 17b that block access of the fusion peptide to the target cell by binding at the apex of Env, and antibodies such as 8066 [55], D5 [56] or HK20 [57] that bind Env following its activation, but before fusion between viral and target cell membranes.

## Supporting Information

Figure S1 **3D visualization of viruses in tomogram.** (a) Aligned and reconstructed image stacks can be used to create a 3D image of individual viruses frozen in a near-native state. Rendering of the tomographic volume allows visualization of individual Env spikes (red) on the surface of the viral membrane (green) shown “emerging” from a tomographic slice (adapted from [60]). (b) Schematic rendering of trimeric Env spikes displayed on the surface of the virion. (c, d) Slices through reconstructed cryo-electron tomograms of HIV-1 BaL complexed with 17b antibody (c) or sCD4 (d). Peripheral Env glycoprotein spikes are visible on the viral membrane. Scale bar is 100 nm.
(TIF)

Figure S2 **Evidence of 3-foldedness in early stages of refinement before imposition of symmetry.** (a) Schematic showing the strategy used to visualize the data by slicing the map at varying heights along the spike axis. (b) 4.1 Å thick sections through density maps corresponding to each of the ten class averages obtained after the initial classification of the VRC03-bound HIV-1 BaL dataset. Each column represents a single class (numbered from 0 to 9). The red outline indicates the most trimeric class (number 5) and the green outline represents additional classes that were used for the next refinement stage (1,2,3,5,6,7, and 9). (c) Quantitative measurement of 3-foldedness for each class average shown in (b) obtained by measuring the dissimilarity of each class with a 60-degree rotated copy of itself around the 3-fold axis.
(TIF)

Figure S3 **Quantitative evaluation of coordinate fits to density maps derived by sub-volume averaging.** The plots show the landscape of correlation coefficients for fits between the coordinates and the experimentally derived density maps as a function of orientation. The landscapes are shown as 3D surfaces along which correlation coefficients are constant, with levels selected to show both the general shape (blue) and the location of the peak (red) that corresponds to the global maximum for each of the fits. The orientation corresponding to the fits reported in the manuscript are represented at the center of each volume coinciding with the location of the red peaks. The three axes represent the Euler angles needed to sample the entire rotational space in 3D on a uniform grid with 3-degree increments. Each point in the grid represents a distinct rotation in 3D of the X-ray coordinates with respect to the map being fit. Correlation coefficients were computed between the X-ray coordinates at each of these orientations and the density map, ensuring that the spatial displacement for each orientation was optimized. Landscapes corresponding to the fit of the VRC01, VRC02 IgG, VRC02 Fab, and VRC03 IgG-bound states are shown in (a), (b), (c) and (d) respectively.
(TIF)

Figure S4 **Density maps of Env bound to VRC02.** (a–d) Top and side views, respectively, of density maps of native, trimeric HIV-1 BaL Env bound to VRC02 Fab (a, b) or VRC02 IgG (c, d). (e, f) Maps resulting from binding of whole VRC02 antibody or VRC02 Fab are superimposable, as seen in the superposition of the two maps in top and side views, respectively. The VRC02 Fab-bound density map is shown in blue and the whole VRC02-antibody-bound map is shown in green. Coordinates shown are for VRC01-bound gp120 (PDB: 3NGB), with gp120 in red and VRC01 in blue.
(TIF)

Figure S5 **Binding of VRC01, VRC02, or VRC03 result in similar Env quaternary states.** (a, b) Top and side views, respectively, of the superposition of the VRC01- (blue), VRC02- (green) and VRC03-bound (grey) Env maps. The maps are shown with the fits obtained for the VRC01-bound map, fitted with coordinates for gp120-VRC01 Fab (PDB ID:3NGB). Coordinates show gp120 (red) and VRC01 (blue).
(TIF)

Figure S6 **Comparison of initial and final 3D structures of the gp140-17b complex.** (a, b) Re-projections of the density maps from an early stage of refinement (a) and from the final map (b), as presented in Figures 9 and 10. The re-projections from the initial model reflect the views seen in the 2D class averages, providing an independent validation for the model, which was then progressively refined to reveal the greater structural detail shown in (b). (c) Fourier shell correlation plot for the final map of the gp140-17b complex. The map is more ordered in the central portion where the gp41 helices are resolved and the plot reflects contributions from both the more-ordered (central gp41) and less well-ordered (peripheral gp120) regions of the map. Structural information in the map extends to resolutions well beyond ∼9 Å.
(TIF)
