# Supplementary figures and images for: Structural Mechanism of Trimeric HIV-1 Envelope Glycoprotein Activation
Source: PLoS Pathog. 2012 Jul 12;8(7):e1002797. doi: 10.1371/journal.ppat.1002797 (PMC3395603; doi:10.1371/journal.ppat.1002797)

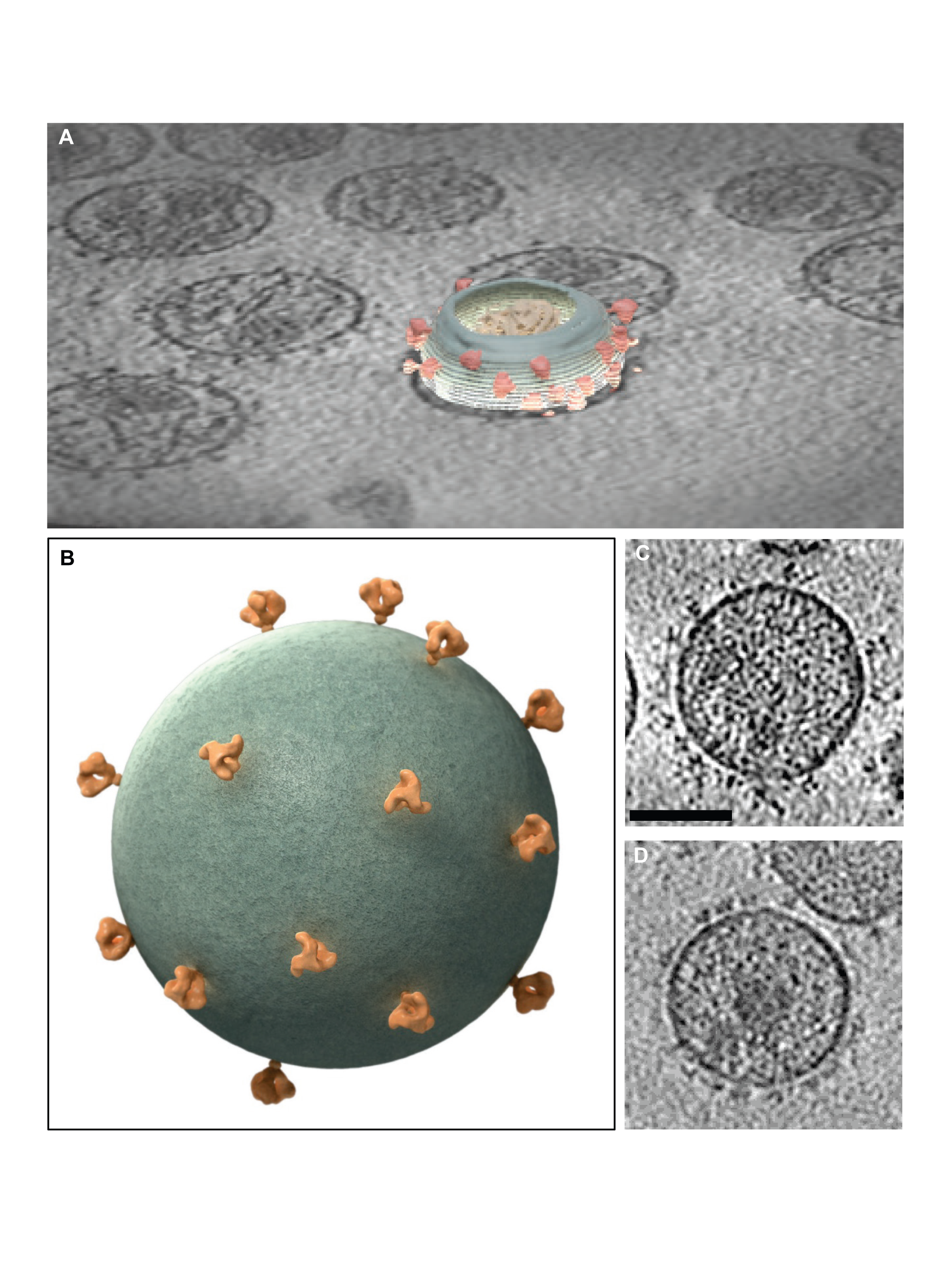

Supplement: Figure S1 — 3D visualization of viruses in tomogram. (a) Aligned and reconstructed image stacks can be used to create a 3D image of individual viruses frozen in a near-native state. Rendering of the tomographic volume allows visualization of individual Env spikes (red) on the surface of the viral membrane (green) shown “emerging” from a tomographic slice (adapted from [60]). (b) Schematic rendering of trimeric Env spikes displayed on the surface of the virion. (c, d) Slices through reconstructed cryo-electron tomograms of HIV-1 BaL complexed with 17b antibody (c) or sCD4 (d). Peripheral Env glycoprotein spikes are visible on the viral membrane. Scale bar is 100 nm. (TIF) [file ppat.1002797.s001.tif]

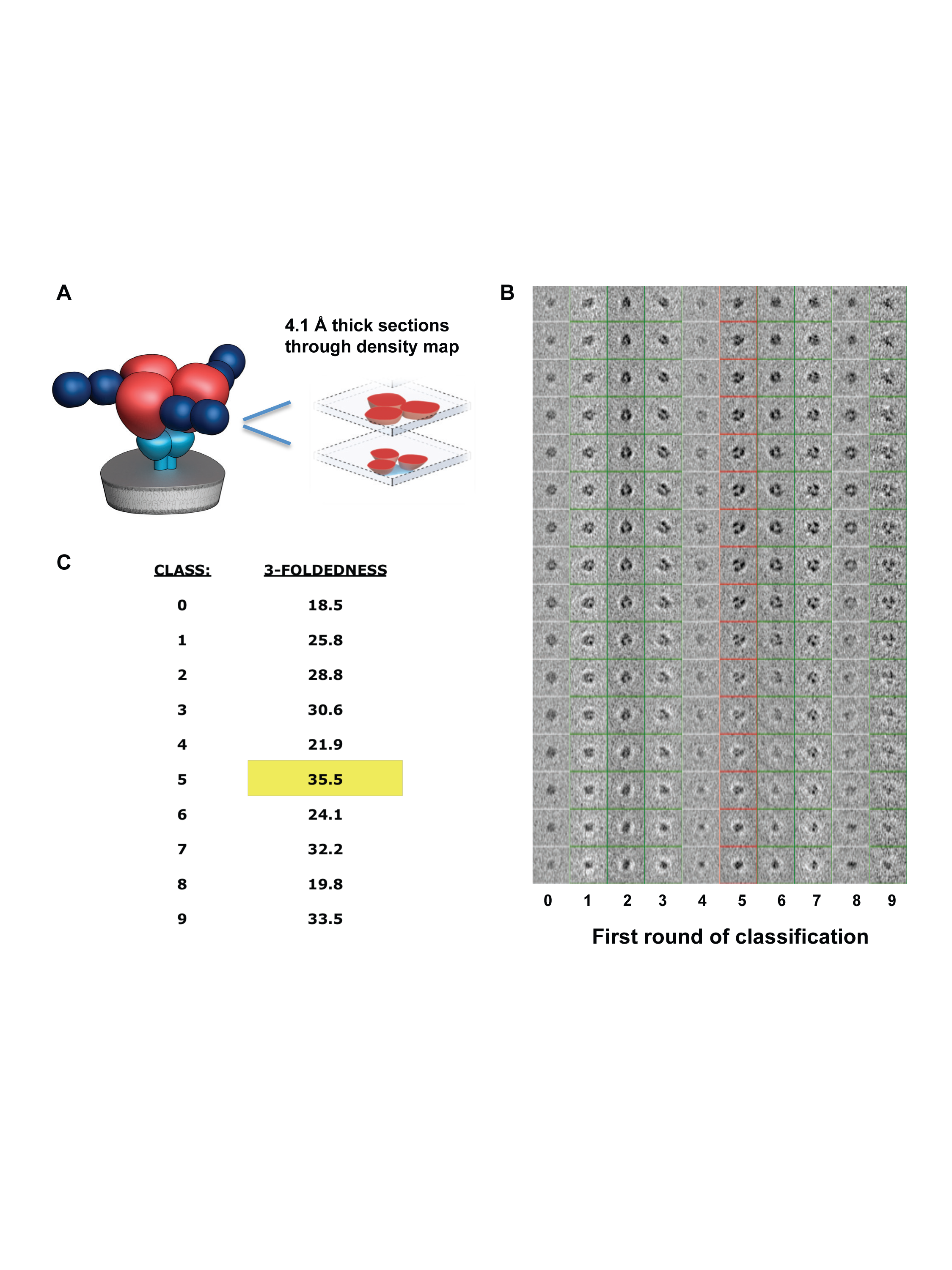

Supplement: Figure S2 — Evidence of 3-foldedness in early stages of refinement before imposition of symmetry. (a) Schematic showing the strategy used to visualize the data by slicing the map at varying heights along the spike axis. (b) 4.1 Å thick sections through density maps corresponding to each of the ten class averages obtained after the initial classification of the VRC03-bound HIV-1 BaL dataset. Each column represents a single class (numbered from 0 to 9). The red outline indicates the most trimeric class (number 5) and the green outline represents additional classes that were used for the next refinement stage (1,2,3,5,6,7, and 9). (c) Quantitative measurement of 3-foldedness for each class average shown in (b) obtained by measuring the dissimilarity of each class with a 60-degree rotated copy of itself around the 3-fold axis. (TIF) [file ppat.1002797.s002.tif]

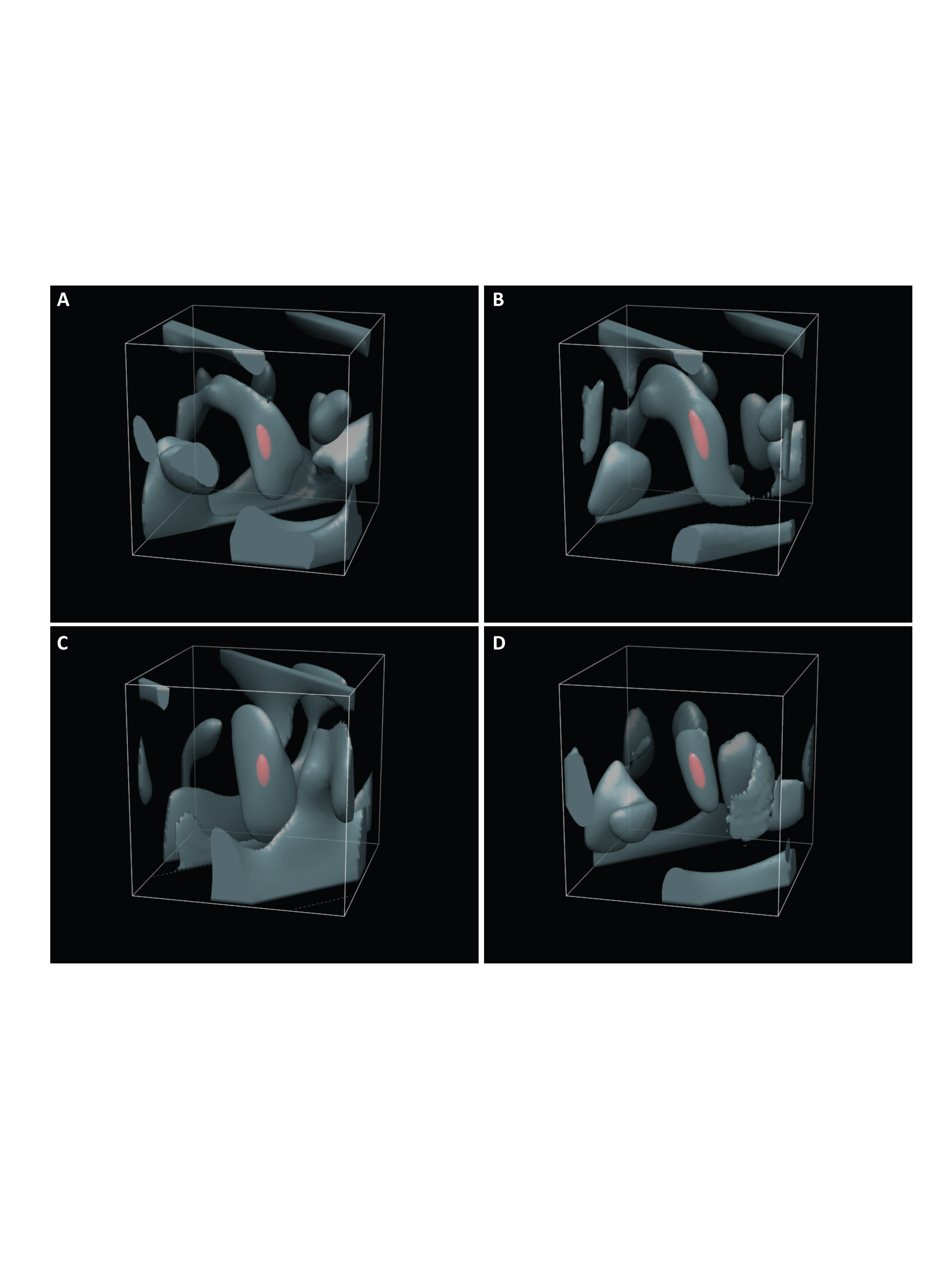

Supplement: Figure S3 — Quantitative evaluation of coordinate fits to density maps derived by sub-volume averaging. The plots show the landscape of correlation coefficients for fits between the coordinates and the experimentally derived density maps as a function of orientation. The landscapes are shown as 3D surfaces along which correlation coefficients are constant, with levels selected to show both the general shape (blue) and the location of the peak (red) that corresponds to the global maximum for each of the fits. The orientation corresponding to the fits reported in the manuscript are represented at the center of each volume coinciding with the location of the red peaks. The three axes represent the Euler angles needed to sample the entire rotational space in 3D on a uniform grid with 3-degree increments. Each point in the grid represents a distinct rotation in 3D of the X-ray coordinates with respect to the map being fit. Correlation coefficients were computed between the X-ray coordinates at each of these orientations and the density map, ensuring that the spatial displacement for each orientation was optimized. Landscapes corresponding to the fit of the VRC01, VRC02 IgG, VRC02 Fab, and VRC03 IgG-bound states are shown in (a), (b), (c) and (d) respectively. (TIF) [file ppat.1002797.s003.tif]

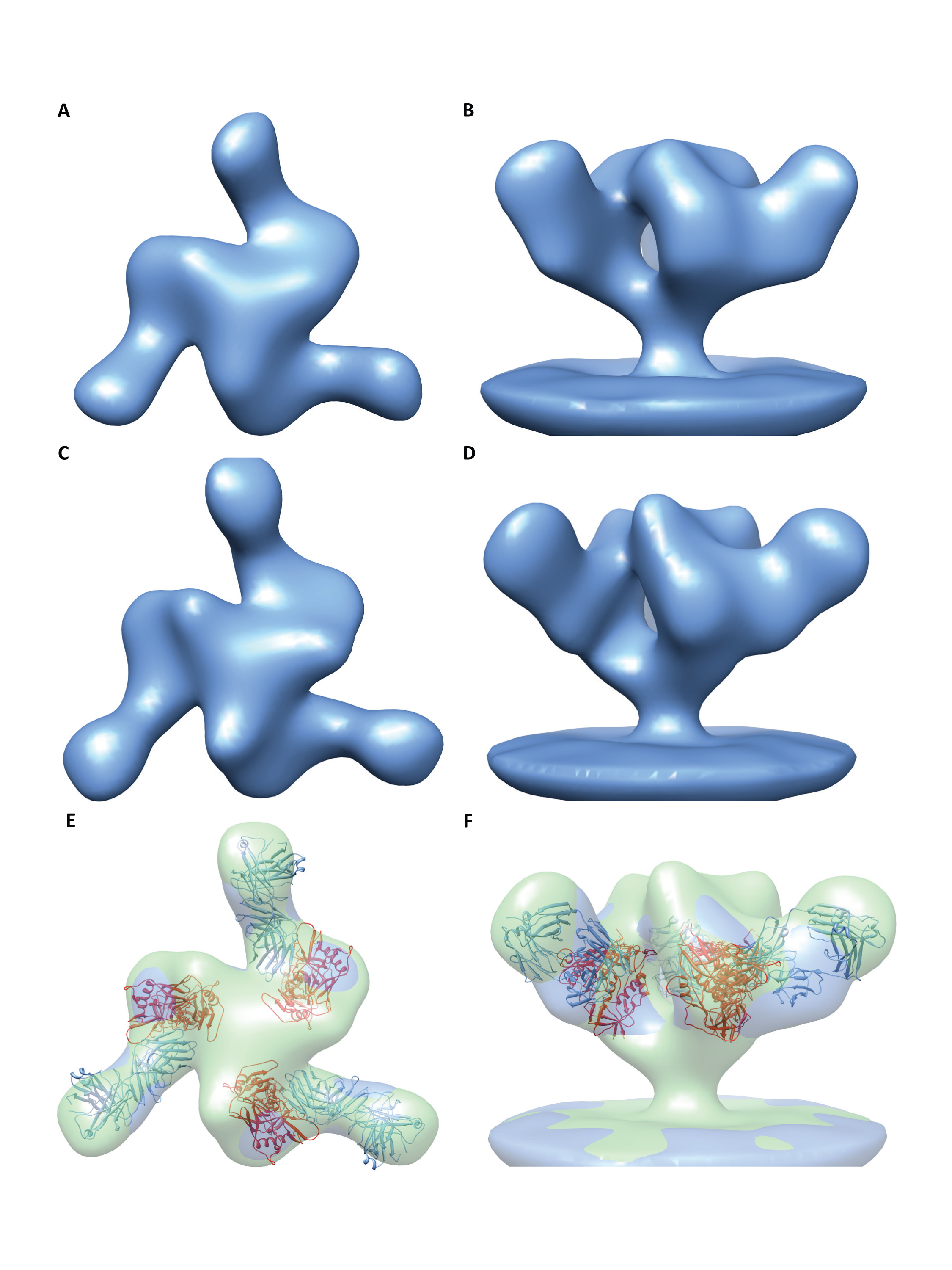

Supplement: Figure S4 — Density maps of Env bound to VRC02. (a–d) Top and side views, respectively, of density maps of native, trimeric HIV-1 BaL Env bound to VRC02 Fab (a, b) or VRC02 IgG (c, d). (e, f) Maps resulting from binding of whole VRC02 antibody or VRC02 Fab are superimposable, as seen in the superposition of the two maps in top and side views, respectively. The VRC02 Fab-bound density map is shown in blue and the whole VRC02-antibody-bound map is shown in green. Coordinates shown are for VRC01-bound gp120 (PDB: 3NGB), with gp120 in red and VRC01 in blue. (TIF) [file ppat.1002797.s004.tif]

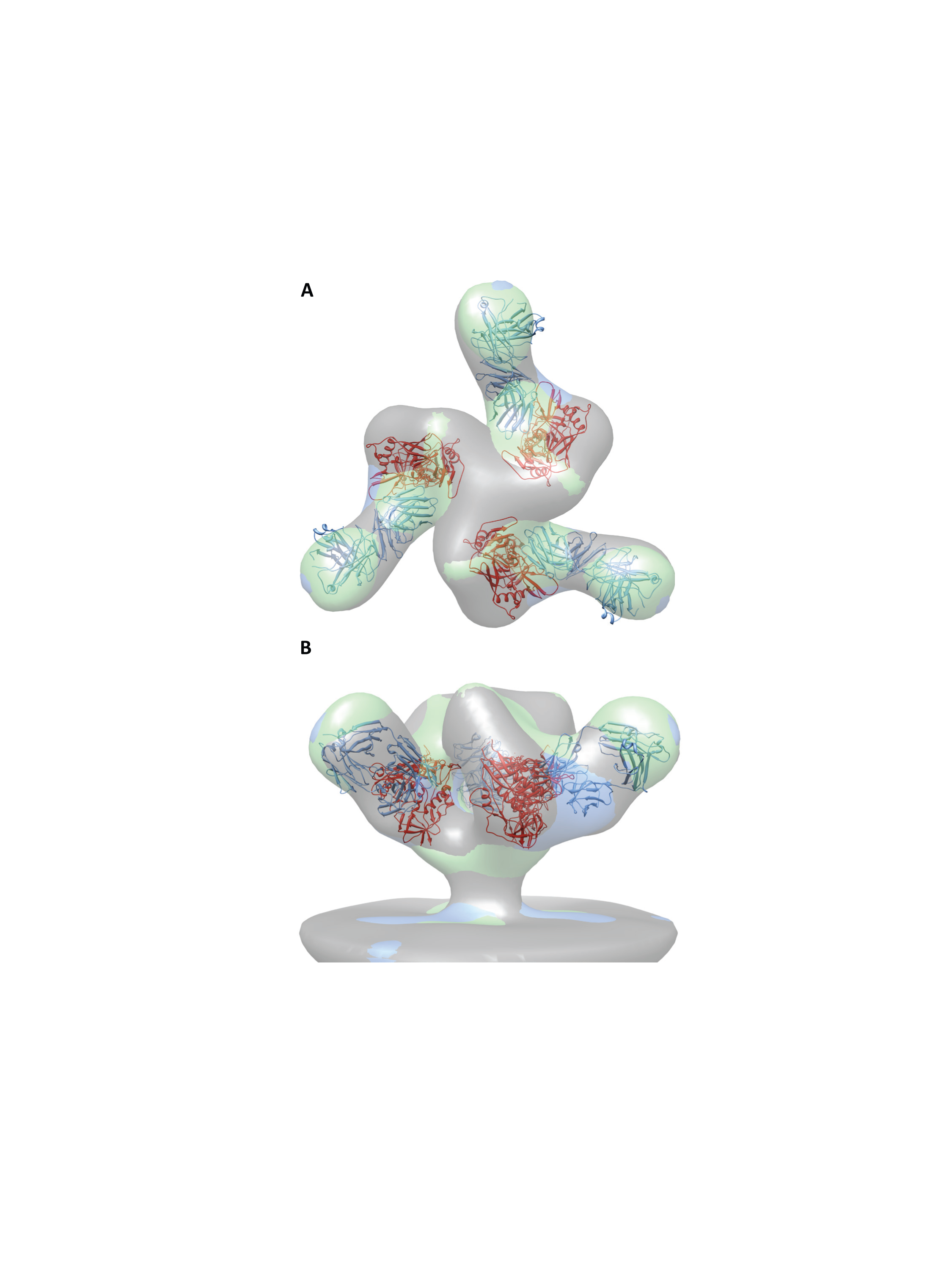

Supplement: Figure S5 — Binding of VRC01, VRC02, or VRC03 result in similar Env quaternary states. (a, b) Top and side views, respectively, of the superposition of the VRC01- (blue), VRC02- (green) and VRC03-bound (grey) Env maps. The maps are shown with the fits obtained for the VRC01-bound map, fitted with coordinates for gp120-VRC01 Fab (PDB ID:3NGB). Coordinates show gp120 (red) and VRC01 (blue). (TIF) [file ppat.1002797.s005.tif]

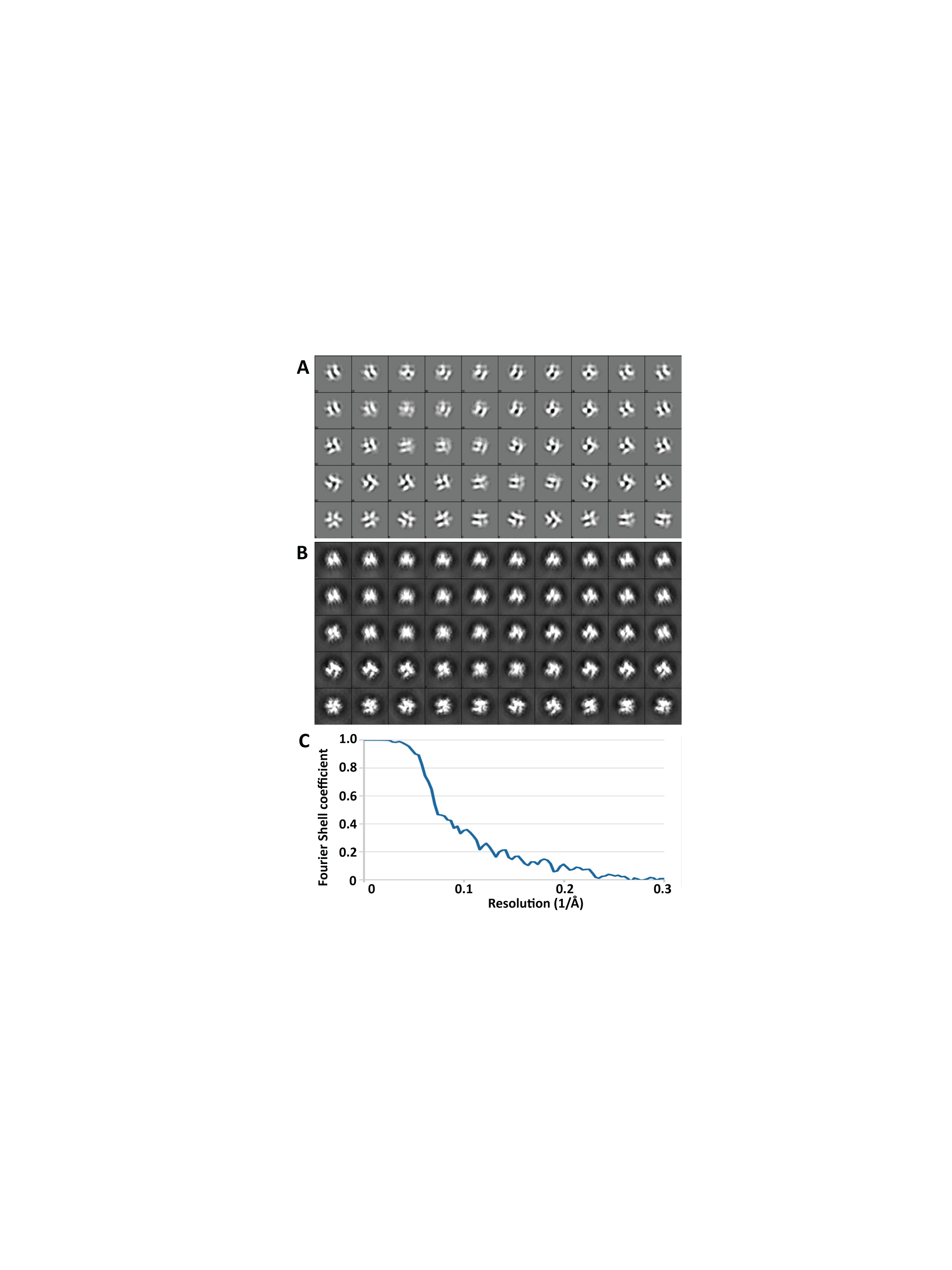

Supplement: Figure S6 — Comparison of initial and final 3D structures of the gp140-17b complex. (a, b) Re-projections of the density maps from an early stage of refinement (a) and from the final map (b), as presented in Figures 9 and 10. The re-projections from the initial model reflect the views seen in the 2D class averages, providing an independent validation for the model, which was then progressively refined to reveal the greater structural detail shown in (b). (c) Fourier shell correlation plot for the final map of the gp140-17b complex. The map is more ordered in the central portion where the gp41 helices are resolved and the plot reflects contributions from both the more-ordered (central gp41) and less well-ordered (peripheral gp120) regions of the map. Structural information in the map extends to resolutions well beyond ∼9 Å. (TIF) [file ppat.1002797.s006.tif]
